# Clinical effects of a standardized Chinese herbal remedy, Qili Qiangxin, as an adjuvant treatment in heart failure: systematic review and meta-analysis

**DOI:** 10.1186/s12906-016-1174-1

**Published:** 2016-07-11

**Authors:** Jin Sun, Kang Zhang, Wen-Jing Xiong, Guo-Yan Yang, Yun-Jiao Zhang, Cong-Cong Wang, Lily Lai, Mei Han, Jun Ren, George Lewith, Jian-Ping Liu

**Affiliations:** Center for Evidence-Based Chinese Medicine, Beijing University of Chinese Medicine, Beijing, China; Complementary and Integrated Medicine Research Unit, Primary Care and Population Sciences, University of Southampton, Southampton, UK; National Research Centre in Complementary and Alternative Medicine (NAFKAM), Faculty of Health Sciences, UiT The Arctic University of Norway, Tromsø, Norway

**Keywords:** Qili Qiangxin capsule, Chinese herbal medicine, Heart failure, Randomized clinical trials, Systematic review, Meta-analysis

## Abstract

**Background:**

Qili Qiangxin capsule is a standardized Chinese herbal treatment that is commonly used in China for heart failure (HF) alongside conventional medical care. In 2014, Chinese guidelines for the treatment of chronic HF highlighted Qili Qiangxin capsules as a potentially effective medicine. However, there is at present no high quality review to evaluate the effects and safety of Qili Qiangxin for patients with HF.

**Methods:**

We conducted a systematic review and meta-analysis and followed methods described in our registered protocol [PROSPERO registration: CRD42013006106]. We searched 6 electronic databases to identify randomized clinical trials (RCTs) irrespective of blinding or placebo control of Qili Qiangxin used as an adjuvant treatment for HF.

**Results:**

We included a total of 129 RCTs published between 2005 and 2015, involving 11,547 patients, aged 18 to 98 years. Meta-analysis showed no significant difference between Qili Qiangxin plus conventional treatment and conventional treatment alone for mortality (RR 0.53, 95 % CI 0.27 to 1.07). However, compared with conventional treatment alone, Qili Qiangxin plus conventional treatment demonstrated a significant reduction in major cardiovascular events (RR 0.46, 95 % CI 0.34 to 0.64) and a significant reduction in re-hospitalization rate due to HF (RR 0.49, 95 % CI 0.38 to 0.64). Qili Qiangxin also showed significant improvement in cardiac function measured by the New York Heart Association scale (RR 1.38, 95 % CI 1.29 to 1.48) and quality of life as measured by Minnesota Living with Heart Failure Questionnaire (MD −8.48 scores, 95 % CI −9.56 to −7.39). There were no reports of serious adverse events relating to Qili Qiangxin administration. The majority of included trials were of poor methodological quality.

**Conclusions:**

When compared with conventional treatment alone, Qili Qiangxin combined with conventional treatment demonstrated a significant effect in reducing cardiovascular events and re-hospitalization rate, though not in mortality. It appeared to significantly improve quality of life in patients with HF and data from RCTs suggested that Qili Qiangxin is likely safe. This data was drawn from low quality trials and the results of this review must therefore be interpreted with caution. Further research is warranted, ideally involving large, prospective, rigorous trials, in order to confirm these findings.

**Electronic supplementary material:**

The online version of this article (doi:10.1186/s12906-016-1174-1) contains supplementary material, which is available to authorized users.

## Background

Heart failure (HF) is a serious and increasingly prevalent worldwide public health problem and has become a major cause of mortality and morbidity [1]. HF is the most common cause of hospitalization in people aged 65 and older [2] and survival rates are reportedly worse than in cancer [3]. According to the European Society of Cardiology (ESC), approximately 26 million people worldwide suffer from HF and which affects 10 % of people over the age of 70, a prevalence which is expected to rise in coming years.

HF is a complex clinical syndrome that results from any structural or functional impairment of ventricular filling or ejection of blood from the heart. Cardinal manifestations include dyspnea and fatigue, which can limit exercise tolerance as well as create fluid retention, and which may lead to pulmonary and peripheral edema [4]. Furthermore, quality of life (QOL) for patients can be adversely impacted owing to sleep-disordered breathing, cognitive dysfunction and neuropsychological disturbances. Conventional medical care for HR typically involves oxygen therapy, diet, diuretics, angiotensin-converting enzyme inhibitors (ACEI) or beta-blockers [5]. Previous research has estimated that the total estimated direct and indirect cost of HF in the US in 2005 was approximately $27.9 billion, with approximately $2.9 billion alone being spent annually on drugs [6]. Despite this significant annual spend, HF continues to be associated with poor prognosis, with absolute mortality rate remaining approximately 50 % within 5 years of initial diagnosis [7]. It is clear that the effectiveness of available care is limited and which warrants further research into optimizing current treatments.

In the recent 2014 Chinese guidelines for treatment of chronic HR, a Chinese herbal remedy, Qili Qiangxin capsules, was mentioned as a potentially effective treatment [8]. Qili Qiangxin capsule is a standardized Chinese herbal treatment that is widely used in China for HF patients and which is frequently administered alongside conventional medical care. It is prepared from 11 Chinese herbs including *astragali radix, ginseng radix et rhizoma, aconite lateralis radix preparata, salvia miltiorrbiza radix et rhizoma, semen descurainiae lepidii, alismatis rhizoma, polygonati odorati rhizoma, cinnamomi ramulus, carthami flos, periploca cortex, and citri reticulatae pericarpium.* Previous research has suggested that Qili Qiangxin may have a role in the treatment of HF through a number of different mechanisms, for example reducing N-terminal pro-brain natriuretic peptide (NT-proBNP), high levels of which are associated with cardiac ventricular volume and pressure overload [9]. In a study on rats with myocardial infarction (MI), Qili Qiangxin induced heart muscle regeneration and improved cardiac function through regulating the balance between tumor necrosis factor (TNF)-α and interleukin (IL)-10, factors closely associated with inflammatory processes in HF [10]. This suggests promise in this area of research which may be of significant interest to the international medical community and which warrants a robust review of the current evidence to date. A number of systematic reviews have been published in this area. However, all have been published in Chinese and have various shortcomings such as insufficient searches, inappropriate outcome selection and lack of quality assessment, leading us to question the scientific rigor of the results and subsequent recommendations [11–16]. The aim of this study was to evaluate the effects and safety of Qili Qiangxin for patients with HF by conducting a systematic review and meta-analysis.

## Methods

The method used to conduct this systematic review has been previously published in a registered protocol [PROSPERO registration: CRD42013006106]. This review was constructed using the PRISMA guidelines (Additional file 1).

### Search strategy

The following electronic databases were searched from date of inception to March 2015: PubMed, the Cochrane Central Register of Controlled Trials (CENTRAL) in the Cochrane Library (Issue January, 2015), China National Knowledge Infrastructure (CNKI), VIP Database, Sino-Med Database, and Wanfang Database. We used the following search terms: (“Qili Qiangxin” OR “qiliqiangxin” OR “qiangxinli”) and (“heart failure” OR “cardiac failure” OR “heart decompensation”). We searched for trials from mainstream registries including Current Controlled Trials (http://www.controlled-trials.com), the World Health Organization International Clinical Trials Registry Platform (WHO ICTRP; http://apps.who.int/trialsearch/), ClinicalTrials.gov trials registry (http://www.clinicaltrials.gov), the Australian New Zealand Clinical Trials Registry (http://www.anzctr.org.au), and Centre Watch (http://www.centerwatch.com). We also hand-searched the reference lists of all full text papers for additional relevant reports. No language restrictions were imposed.

### Inclusion criteria

We accepted RCTs regardless of blinding procedures and included only parallel design studies. Only human studies were included in this review and we required the use of internationally-accepted criteria for diagnosis of HF. We placed no other requirements of the participant population in terms of gender, age, etiology, ethnic group, severity or course of disease. Only Qili Qiangxin capsules, composed of the aforementioned 11 Chinese herbs, were accepted as the intervention. This could be used alone or alongside appropriate control treatments such as placebo, conventional treatment or no treatment, and trials with any other Chinese herbal medicine in control group will be excluded. Our primary outcome measures were all-cause mortality or cardiovascular mortality due to HF, major cardiovascular events such as MI, outpatient visits, hospitalizations or re-admission for HF. Secondary outcomes measures were quality of life (QOL) measured by Minnesota Living with Heart Failure Questionnaire (MLHFQ), New York Heart Association (NYHA) functional classification, echocardiography measurements, six-minutes walking distance (6MWD), plasma amino-terminal pro-brain natriuretic peptide (NT-pro-BNP). We also collected safety and adverse events data. We included only RCTs reporting one or more of these outcomes.

### Data extraction and quality assessment

Two authors (from J Sun, K Zhang, WJ Xiong, and YJ Zhang) independently identified articles for eligibility with any disagreements resolved through discussion with a third party (JP Liu). Two authors independently extracted data and which included patient characteristics, details of the intervention and control groups, outcome measures and main results. The same process was used to assess the methodological quality of included RCTs using the risk of bias tool as described in the Cochrane Handbook for Systematic Reviews of Interventions [17]. This process requires seven criteria to be assessed: random sequence generation, allocation concealment, blinding of participants and personnel, blinding of outcome assessors, incomplete outcome data, free of selective reporting, and other bias.

### Strategy for data synthesis

Statistical analyses were performed by using RevMan 5.2 software (The Cochrane Collaboration). Pooled risk ratio (RR) with 95 % confidence interval (CI) of dichotomous outcomes was used to estimate report effect. Continuous data was presented as mean difference (MD) with 95 % CI. We used a fixed effect model unless there was evidence of heterogeneity. Heterogeneity was assessed using both the Chi-squared test and the I-squared statistic, and we considered an I-squared value greater than 50 % to be indicative of substantial heterogeneity. If missing data from the original trials are available, intention-to-treat analysis will be applied for primary outcome.

## Results

### Description of studies

**Fig. 1:**
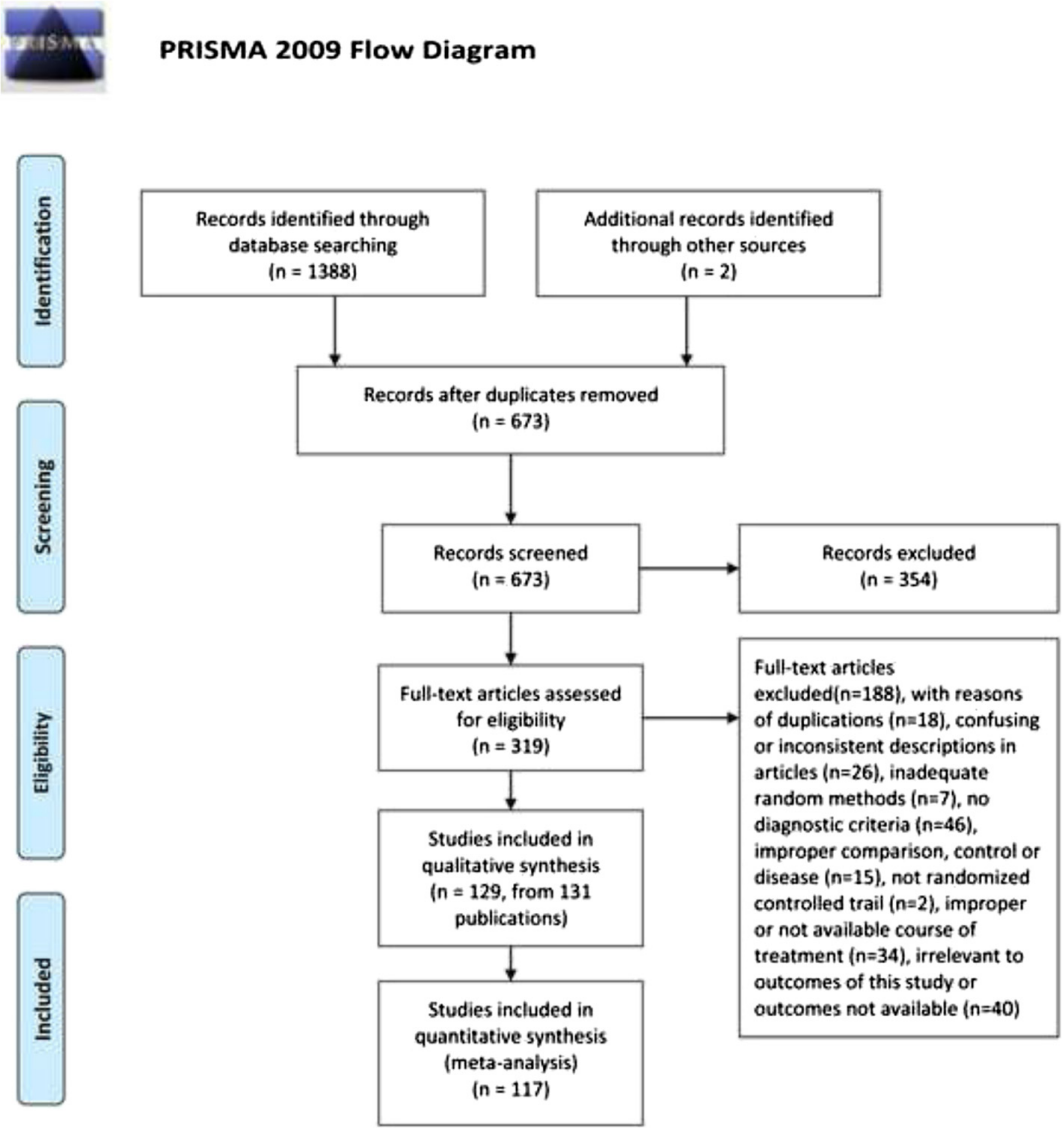
Flow chart of study searching and selection

Our search in March 2015 from six databases and other sources identified 1,390 potentially eligible articles. After removing duplicates and reviewing full text articles, we eventually included 129 randomized clinical trials published as 131 articles (Fig. 1). All 129 studies were included in our qualitative synthesis and of these, 117 studies were eligible for our meta-analysis.

### Study characteristics

Included trials were published between 2005 and 2015 with a larger proportion of trials published in 2013 (*n* = 41 trials, 31.78 %) and 2012 (*n* = 23 trials, 17.83 %). The 129 trials involved 11,547 patients diagnosed with HF with an age range of 18 to 98 years. Sixteen trials enrolled only elderly patients over the age of 60 (*n* = 16, 12.40 %). The majority of trials included both male and female patients (*n* = 127 trials, 98.45 %). Two trials included only male patients (Wei XB 2013, Miao S 2013); two trials provided gender details only for participants who completed the trial (Li XL 2013, Kuang JB 2008). 58.23 % of total populations were male except one trial failed to provide gender detail (Tao X 2011).

Included trials used the following diagnostic criteria: American College of Cardiology/American Heart Association (ACC/AHA) guidelines, European Society of Cardiology (ESC) guidelines, World Health Organization/International Society and Federation of Cardiology (WHO/ISFC) guidelines, World Health Organization/International Society of Hypertension (WHO/ISH) guidelines, Chinese Medical Association (CMA) guidelines, guidelines issued by Ministry of Health (MoH) in China, Framingham criteria, or textbook criteria which were consistent with internationally-used diagnostic criteria .

One hundred seventeen trials (s1-s118, Additional file 2) involving 10,170 patients compared Qili Qiangxin capsules plus conventional treatment to conventional treatment alone. Three trials (s119-s121, Additional file 2) involving 702 patients compared Qili Qiangxin capsule plus conventional treatment to placebo plus conventional treatment. Nine trials (s122-s131, Additional file 2) included 675 patients comparing Qili Qiangxin capsules plus conventional treatment to recommended pharmaceuticals (including thiazide diuretics, benazepril, captopril, metoprolol, irbesartan, hydrochlorothiazide and digoxin plus conventional treatment). Almost all patients in the included trials presented with comorbidities such as hypertension, hypotension, valvular heart disease, pulmonary heart disease, cardiomyopathy, diabetes mellitus, chronic renal insufficiency, coronary heart disease, acute MI and sinus bradycardia. The duration of treatment ranged from 1 to 12 months, and the most commonly adopted was 1 month (*n* = 54 trials). Nineteen trials had 6 or more months of treatment.

The main outcome in the majority of trials was reported to be left ventricular ejection fraction (LVEF) (*n* = 91 trials). Other primary outcomes adopted included mortality (*n* = 6 trials), major cardiovascular events (*n* = 3 trials) or re-hospitalization (*n* = 9 trials). Details of each study are listed in Table 1.

**Table 1:** Characteristics of included studies

ID (Author year)	Disease	Setting (in/out patients)	Diagnosis criteria	Sample size	Age-T	Age-C	Men-%	Course of treatment
Bai LQ 2013	HF	in	NYHA	40	45 ± 3.7	52 ± 4.2	57.5	3M
Cai RF 2013	CHF	NA	CMA 2009; MoH 2002; Framingham criteria; NYHA	50	52-80	52-81	54	4W
Cai YP 2013	CHF	in or out	Framingham criteria; NYHA	108	73.2 ± 11.5		60.19	6M
Chen L 2009	CHF	in	ACC/AHA 1995; MoH 2002; NYHA	61	67.2 ± 11.5	63.1 ± 13.8	57.38	4W
Chen TC 2013	CHF	NA	CMA 2007; NYHA	52	71-92	68-91	53.85	4W
Chen WQ 2012	CHF	in or out	CMA 2007; NYHA	120	58 ± 14	57 ± 14	59.17	6M
Chen XH 2014	HF	NA	CMA 2014	60	63 ± 10	62 ± 11	52.17	6M
Cheng XD 2013	ischemic heart failure	in	ACC/AHA criteria; NYHA	90	56.4 ± 6.2	54.2 ± 5.9	55.56	6W
Cui LL 2012	CHF	in	CMA 2007; NYHA	68	59.41 ± 9.68	58.6 ± 7.71	48.53	5M
Dai JX 2013	chronic congestive heart failure	NA	*Guidelines for the Diagnosis and Treatment of Cardiovascular Disease* 2006	100	70.2		58	4W
Ding LB 2010	HF	in	*Internal Medicine* 2004; MoH 2002	43	45 ± 12.2	42 ± 11.3	55.81	2M
Ding SY 2013	CHF	NA	*Practical Internal Medicine* 2001; NYHA	72	48-82	51-79	45.83	4W
Dong MX 2013	chronic congestive heart failure	in	*Practical Internal Medicine* 2001; NYHA	114	71.5		59.65	4W
Du YK 2014	diastolic heart failure	out	CMA 2007	102	60-86	59-82	39.22	30D
Duan JH 2010	CHF	NA	*Clinical Cardiology* 1996	61	63.4 ± 10.3	61.5 ± 8.7	72.13	3M
Fan J 2013a	CHF	NA	*Internal Medicine* 2008; NYHA	86	68.1 ± 9.5	68.7 ± 9.6	55.81	2M
Feng QT 2013	chronic congestive heart failure	out	Framingham criteria	42	55.6 ± 10.32	54.66 ± 10.41	59.52	6M
Fu JZ 2012	CHF		*Internal Medicine* 2004; NYHA	60	77.3 ± 10.2	78.0 ± 10.1	58.33	3M
Gao JB 2011	chronic congestive heart failure	in or out	MoH 2002	167	58 ± 11		58.08	4W
Gu XM 2009	CHF	in or out	AHA criteria	38	52 ± 9	48 ± 10.6	68.42	2M
Gu XM 2013	CHF	in or out	ACC/AHA 2005; NYHA	65	57 ± 18.5	56 ± 16	66.15	4W
Gu YY 2012	CHF	in or out	NYHA	100	65.5 ± 10.1	62.3 ± 12.5	58.57	6M
Guan SY 2012	CHF	NA	Consensus for diagnosis and treatment of heart failure with normal ejection fraction in China 2010	82	56 ± 13	54 ± 13	42.68	8W
Guan SY 2013	CHF	NA	CMA 2007	72	55 ± 12	54 ± 13	52.78	12W
Guo P 2014	CHF	NA	ACC/AHA criteria; NYHA	90	71.6 ± 4.5	72 ± 3.5	58.7	3M
Guo SL 2011	chronic congestive heart failure	in	CMA 2007	120	60-80		60	3M
Guo WB 2013	chronic congestive heart failure	in	CHF criteria 1979	70	61.5 ± 9.12	57.4 ± 8.97	57.14	8W
Hu B 2013	CHF	in or out	Framingham criteria	80	39-75	36-76	78.75	3M
Huang B 2010	chronic congestive heart failure	NA	Framingham criteria; NYHA	100	61.2 ± 11.8	60.8 ± 12.5	60	6M
Huang YQ 2012	chronic congestive heart failure	NA	*Internal Medicine* 2004	46	60 ± 7.5	58 ± 7.8	47.83	4W
Huang Z 2014	CHF	in or out	CMA 2007; NYHA	60	35-74	35-74	58.33	12W
Jin Y 2012	heart failure derived from ischemic cardiomyopathy	NA	ICM criteria (Felker GM) 2002; NYHA	100	62.0 ± 15.2	63.0 ± 13.9	47	12W
Jing GJ 2009	CHF	in or out	WHO criteria; CMA 2002; MoH 2002	60	62.0 ± 3.5	63 ± 4.0	58.33	4W
Kuang JB 2008	CHF	NA	ESC criteria	106	71.6		45.16	8W
Li DW 2013	chronic congestive heart failure	in	NYHA	78	66		51.28	12W
Li GM 2011	chronic congestive heart failure	in or out	Framingham criteria; NYHA	120	72.5	71.8	56.67	4W
Li LC 2013	heart failure derived from ischemic cardiomyopathy	NA	NYHA	110	61 ± 13	63 ± 12	54.55	4W
Li P 2011	CHF	in or out	CMA 2002;NYHA	76	66.1 ± 7.8	66.3 ± 7.2	59.21	3M
Li Q 2014	HF	NA	NYHA	120	71.4 ± 8.0	70.2 ± 7.1	70.59	6M
Li RY 2010a	CHF	NA	NYHA	86	68.4 ± 1.3	68.1 ± 1.3	67.44	4W
Li SQ 2014	CHF	in or out	CMA 2007; NYHA	147	41.2 ± 12.5	39.8 ± 13.2	40.82	2M
Li SZ 2009	congestive heart failure	NA	NYHA	39	62 ± 7		56.41	4W
Li T 2010	CHF	in	Boston criteria; NYHA	44	56 ± 14		56.82	4W
Li WY 2013	CHF	NA	*Internal Medicine* 2008; NYHA	90	71 ± 4.6	73 ± 4.2	57.78	4W
Li XL 2013	CHF	NA	CMA 2007; NYHA	512	56.98 ± 11.59	57.53 ± 11.05	75.36	12W
Li YH 2013	CHF	NA	NYHA	80	67.3 ± 11.6		70	3M
Li YX 2012	diastolic heart failure	in or out	ESC criteria; MoH 2002	100	61.6 ± 5.1	61.4 ± 5.4	43	6M
Li YX 2013	CHF	NA	ESC 2007; MoH 2002;*Collateral Disease Theory* 2006	80	61.6 ± 5.1	61.4 ± 5.4	46.25	12M
Lin JH 2008	CHF	in or out	*Clinical Cardiology* 1999; NYHA	80	58 ± 12		60	4W
Lin ZJ 2010	heart failure derived from ischemic cardiomyopathy	NA	WHO/ISFC 1980; NYHA	60	40 ± 13	38 ± 12	45	6M
Liu HL 2008	CHF	NA	*Guidelines for Cardiovascular Disease* 2005; NYHA	86	32.9 ± 4.1	33.1 ± 3.2	52.33	4W
Liu J 2008	heart failure derived from ischemic cardiomyopathy	NA	WHO/ISFC 1980; NYHA	41	41 ± 11	40 ± 11	60.98	6M
Liu LX 2014	HF	NA	*Practical Internal Medicine* 2009	60	64.4 ± 11.5	64.5 ± 11.3	61.67	12W
Liu SJ 2009	CHF	in or out	*Clinical Cardiology* 1996; MoH 2002; NYHA	45	68.2 ± 7.6	66.8 ± 8.2	60	4W
Liu T 2013	HF	NA	NYHA	95	63.7 ± 7.4	65.7 ± 7.6	71.58	4W
Liu TR 2010	CHF	NA	NYHA	84	46-68	45-70	63.1	8W
Liu WJ 2007	ischemic cardiomyopathy	in	Felker's criteria	60	66 ± 10	65 ± 11	70	4M
Liu XC 2008	refractory heart failure	NA	NYHA	120	56-79	58-78	80.83	30D
Liu XC 2011	CHF	in or out	AHA criteria; NYHA	80	56.9 ± 7.3	57.0 ± 7.6	65	8W
Liu XG 2013	CHF	in or out	AHA criteria; NYHA; MoH 2002	60	61.2 ± 11.8	60.8 ± 12.5	58.33	3M
Liu XM 2010	HF	NA	ACC/AHA criteria; NYHA	76	65-82		52.63	3M
Liu XM 2013	CHF	NA	CMA 2007	64	69 ± 11	68 ± 12	57.81	4W
Liu YJ 2012	HF	in or out	*Clinical Cardiology*; NYHA	60	78-90	76-92	68.33	30D
Long F 2009	CHF	in	*Clinical Cardiology* 1996; CMA 2002; NYHA	110	20-73	18-70	53.64	4W
Lu JP 2012	CHF	in	CMA 2007; NYHA	60	73.2 ± 12.5	72.9 ± 11.8	65	24W
Luo Q 2013	chronic congestive heart failure	in	NYHA	60	62.5 ± 13.0	64.5 ± 12.2	56.67	3M
Ma AP 2013	CHF	NA	NYHA	96	66.28 ± 4.92	65.84 ± 5.06	56.25	6M
Ma FF 2008a	CHF	in	Boston criteria 1985; NYHA	120	65.4	64.6	46.67	4W
Ma FF 2008b	CHF	in	Boston criteria 1985; NYHA	65	64.1 ± 17.2		46.15	4W
Ma L 2010	CHF	in or out	ESC 1995; NYHA; MoH 2002	117	52.3 ± 9.2	50.1 ± 10.5	61.54	4W
Ma RX 2014	CHF	in	CMA 2007	120	62 ± 12	60 ± 11	65.75	4W
Miao S 2013	HF	NA	NYHA	102	77.2 ± 6.1		100	2M
Niu LY 2012	CHF	NA	ACC/AHA criteria; NYHA; MoH 2002	60	63.2 ± 4.1	60.8 ± 5.4	63.33	4W
Pang XM 2008	CHF	NA	Framingham criteria	31	66 ± 12		48.39	4W
Qiu X 2013	CHF	NA	NYHA	60	62 ± 4.2	60 ± 3.2	66.67	3M
Rao LZ 2012	CHF	NA	CMA 2007; NYHA	80	65 ± 15	64 ± 14	51.25	4W
Shen R 2010	HF	in	NYHA	62	74 ± 5	74 ± 7	77.42	28D
Shen XR 2014	CHF	in or out	ACC/AHA criteria; NYHA	122	62 ± 6		66.45	12M
Shi CP 2013	CHF	in	ISFC/WHO 1979; NYHA	120	64.5 ± 6.2		67.5	3M
Su HM 2007	chronic congestive heart failure	in	Framingham criteria; NYHA 1994	70	55.7	54.6	60	30D
Su LJ 2012	CHF	in or out	CMA 2002; *Practical Internal Medicine* 2001; NYHA	69	NA	NA	65.22	8W
Su RY 2013	CHF	NA	CMA 2007; NYHA	86	69		46.51	4W
Sun LP 2007	chronic congestive heart failure	in	NYHA	60	62 ± 12		63.33	12W
Tang SY 2013	CHF	in	CMA 2002; *Clinical Cardiology* 1996; *Internal Medicine* 2004; NYHA	80	65.4	64.6	47.5	4W
Tao X 2011	CHF	NA	ESC 2008	100	NA	NA	0	4W
Tian Y 2011	diastolic heart failure	NA	ESC criteria; MoH 2002	100	58.0 ± 8.2	58.0 ± 8.5	47	1M
Wang N 2014	CHF	in or out	MoH 2002	54	55-75	58-76	53.7	8W
Wang Q 2012	CHF	in or out	NYHA	80	40-70	41-70	61.25	24W
Wang SZ 2012	HF	NA	*Diabetic Cardiomyopathy* 2010; NYHA	60	60 ± 13	60 ± 11	61.67	4W
Wang YY 2013	chronic congestive heart failure	in or out	NYHA	79	62.6 ± 2.4	61.4 ± 2.3	53.16	4W
Wei XB 2013	CHF	in	NYHA; ACC/AHA 2009	84	87 ± 6		100	12W
Wen Y 2012	CHF	NA	CMA 2007; NYHA	90	70.4 ± 5.6		57.78	1M
Wu GL 2015	CHF	out	CMA 2007; NYHA	104	67.5 ± 6.8	66.7 ± 7.1	64.32	2M
Wu SP 2014	CHF	in or out	CMA 2014	130	52.2 ± 5.8	53.8 ± 7.3	73.8	4W
Wu Xian 2014	CHF	in	CMA 2007	60	64.47 ± 8.23	63.57 ± 8.94	55.38	4W
Xiong SQ 2014	CHF	in	CMA 2007; NYHA	80	61.2 ± 7.11	62.3 ± 7.45	52.5	6W
Xu GS 2014	CHF	NA	NYHA	64	55.7 ± 14.0	53.6 ± 15.0	59.38	120D
Xue L 2014	CHF	in	A list of clinical manifestation	124	42-86		64.52	8W
Xue LX 2008	chronic congestive heart failure	in or out	AHA criteria	80	56.9 ± 7.3	57.0 ± 7.6	65	8W
Yan KL 2012	CHF	in	*Practical Internal Medicine* 2009; NYHA	120	65.2 ± 17.5		56.67	12W
Yang DK 2014	CHF	in	NYHA	60	66.0 ± 12.06	65.8 ± 11.33	63.33	4W
Yang F 2007	CHF	NA	ESC 2005	128	66.0 ± 14.0	65.0 ± 15.0	56.25	8W
Yang HT 2012	CHF	in	*Clinical Cardiology* 1999; NYHA	100	58.5 ± 7.0	59 ± 7.6	57	4W
Yang HT 2013	CHF	in	*Clinical Cardiology* 1999	100	58.5 ± 7.0	59 ± 7.6	57	4W
Yang J 2013	CHF	NA	CMA 2007; NYHA	90	56.8 ± 4.3	C1 (57.2 ± 4.1) C2 (57.1 ± 3.9)	43.33	12W
Yang W 2012	CHF	in	CMA 2007; NYHA	80	60.52 ± 12.6	62.7 ± 9.6	56.25	4W
Yao L 2011	CHF	in	CMA 2007; NYHA	102	52 ± 11	56 ± 9	52.94	8W
Ye RS 2013	CHF	NA	CMA 2007; NYHA	80	65-92	65-90	56.25	6M
Ye S 2012	CHF	in	CMA 2007; NYHA	114	60.29 ± 5.62		53.51	3M
Yin ZL 2009	congestive heart failure	in	Framingham criteria; NYHA	50	57.4 ± 7.6		66	4W
Ying M 2013	CHF	NA	Framingham criteria; NYHA	80	50-80		52.5	3M
Yu JH 2008	diastolic heart failure	in	Framingham criteria; CHFA 2001; NYHA	70	65.7 ± 6.1	66.1 ± 8.2	60	12M
Yuan JK 2012	HF	in	NYHA	62	41.65 ± 9.33	43.08 ± 7.55	58.06	4W
Zhai N 2015	CHF	NA	CMA 2014	80	70.6 ± 4.4		61.54	12W
Zhang CA 2013	CHF	in or out	AHA 1995; NYHA	83	59.31 ± 10.19	61.0 ± 8.39	66.27	12W
Zhang H 2011	CHF	NA	ISFC/WHO criteria	123	45-80	50-82	60.16	4W
Zhang J 2015	CHF	NA	Practical Internal Medicine 1998,NYHA	60	63.1 ± 9.5	62.5 ± 8.2	50	4W
Zhang R 2014	CHF	in	CMA 2007; NYHA	80	55.0 ± 10.9	53.0 ± 11.3	62.14	8W
Zhang WL 2013	CHF	in	NYHA	94	47-86	45-87	58.51	4W
Zhang XX 2010	left cardiac insufficiency	NA	NYHA	136	53.5		52.94	6M
Zhao JS 2014	CHF	NA	CMA 2007; NYHA; MoH 2002	450	54.8 ± 4.6	55.3 ± 4.7	48.33	12W
Zhao MJ 2009 &Zhao MJ 2012	HF	in or out	Framingham criteria; NYHA	68	46-69	45-69	64.71	4W
Zheng JJ 2012	CHF	in	CMA 2007; NYHA	76	64 ± 15	64 ± 14	55.26	6M
Zheng LW 2013	CHF	in or out	CMA 2007; NYHA	164	66.9 ± 11.5	67.8 ± 12.0	53.05	12W
Zheng WH 2014	refractory heart failure	NA	NYHA	87	63.44 ± 2.20	61.92 ± 2.70	56.32	6W
Zhou FZ 2011a	congestive heart failure	in	NYHA	59	66		69.49	12W
Zhou Y 2013	HF	NA	*Internal Medicine* 2010	60	56.9 ± 7.3	57.0 ± 7.6	63.33	8W
Zhu HG 2012	CHF	in or out	NYHA	78	63.1	63.4	61.54	3M
Zhuo JY 2013	CHF	NA	NYHA	136	62.74 ± 7.78	61.01 ± 8.12	54.41	4W

*ACC* American College of Cardiology, *AHA* American Heart Association, *C* control group, *CHF* chronic heart failure, *CHFA* Chinese Heart Failure Association, *CMA* Chinese Medical Association, *ESC* European Society of Cardiology, *HF* heart failure, *ISFC* International Society and Federation of Cardiology, *ISH* International Society of Hypertension, *MoH* Ministry of Health in China, *NA* not available, *NYHA* New York Heart Association, *T* treatment group, *WHO* World Health Organization

### Methodological quality of included trials

The methodological quality of included trials was generally low as most failed to provide protocols (Additional file 3). Nineteen trials (*n* = 2388 participants, 20.68 %) were judged as low risk on random sequence generation. Of these twenty, 17 trials used random number tables (*n* = 1774 participants, 72.29 %), one trial used statistical software (*n* = 512 participants, 21.44 %) and one trial used drawing lots. Only one (Li XL 2013) trial reported allocation concealment was judged as low risk; one trial (Yang F 2007) described using an envelope but failed to provide further details and was subsequently judged as presenting with unclear risk of bias.

We judged five trials on the use of blinding methods. Of these, three double-blind placebo controlled trials (Yu JH 2008, Li XL 2013, Yu JH 2008) were judged as low risk (*n* = 702 participants, 6.08 %). For the remaining two trials, one single-blind trial (Guo WB 2013) and one double-blind trial (Wei XB 2013) did not mention who was blinded and were judged as unclear risk. Besides, only one trial (Li XL 2013) reported the blinding of outcome assessment (*n* = 512 participants, 4.43 %).

Overall six trials reported patient attrition (*n* = 948 participants, 8.21 %). Of these, three trials (Chen WQ 2012, Fu JZ 2012, Sun LP 2007) reported no drop-out. Three trials (Guo P 2014, Li XL 2013, Kuang JB 2008) described the occurrence of attrition, one (Li XL 2013) used a flow chart to describe patient attrition in the two parallel groups throughout the whole study and adopted intention to treat (ITT) analysis, and one (Kuang JB 2008) was judged as high risk as the authors failed to provide drop-out reasons in text. The remaining trials reported the same number of participants between the baseline and data analyzed, except one study (Guo WB 2013) which reported six cases less than the number at baseline without providing further explanation.

**Fig. 2:**
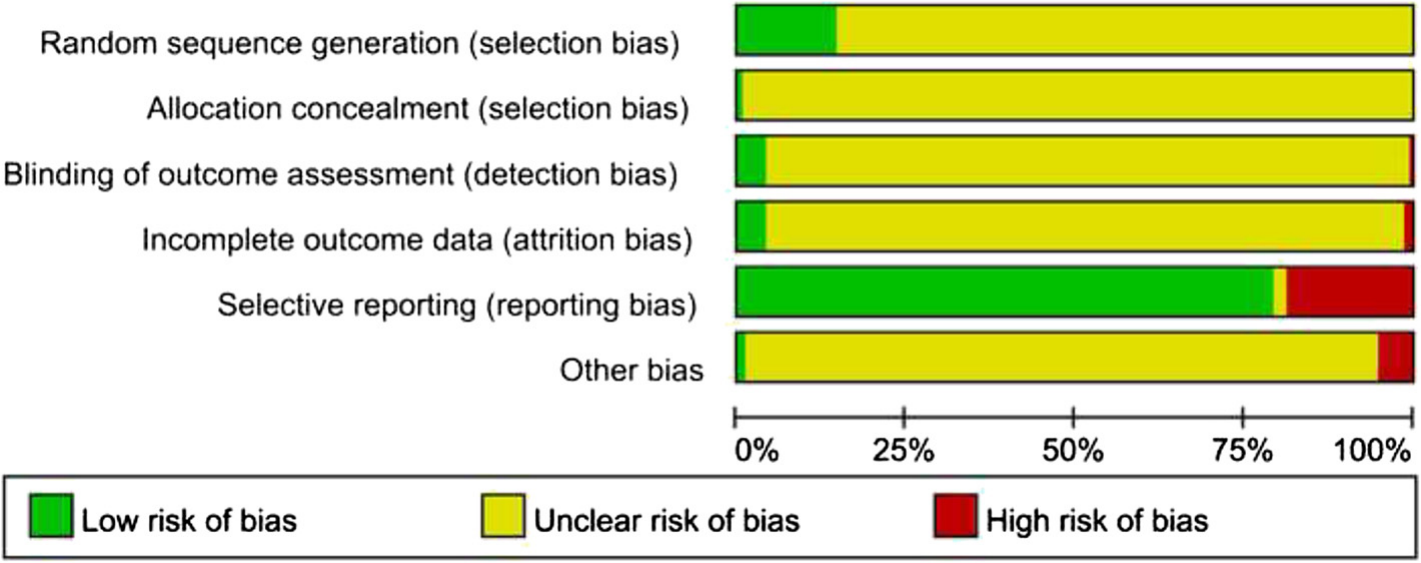
Risk of bias summary

Twenty-four trials failed to report all outcomes listed in the methods. The remaining trials reported all outcomes as specified in methods, although only one provided a study protocol. Insufficient information was available for us to conduct a risk of bias assessment in terms of selective reporting. In terms of other biases, only 122 trials reported comparability of baseline data and only one study (Li XL 2013) reported conducting a sample size calculation (Fig. 2).

### Effects of interventions

#### Qili Qiangxin capsule plus conventional treatment versus conventional treatment

Our meta-analysis (Additional file 4) showed that in comparison to conventional treatment alone, Qili Qiangxin plus conventional treatment did not have a statistically significant effect on reducing mortality (RR 0.53, 95 % CI 0.27 to 1.07, I^2^ = 0 %). Qili Qiangxin plus conventional treatment led to a significant reduction in major cardiovascular events (RR 0.46, 95 % CI 0.34 to 0.64, I^2^ = 0 %; defined as outpatient visits or re-admission for HF and cardiogenic or all-cause mortality), when separately analyzed, it significantly reduced the outpatient visits (*n* = 1 trial, 60 participants, RR 0.22, 95 % CI 0.05 to 0.94), but did not have a statistically significant effect on reducing cardiogenic mortality (*n* = 1 trial, 56 participants, RR 0.67, 95 % CI 0.21 to 2.11). Meta-analysis showed a statistically significant reduction in hospitalizations due to HF (RR 0.49, 95 % CI 0.38 to 0.64, I^2^ = 0 %), and in addition, sensitivity analysis based on consideration of ‘worst-case’ scenarios revealed that missing data did not change the result of this meta-analysis (RR 0.50, 95 % CI 0.39 to 0.65, I^2^ = 0 %). Besides, due to the limited quantity of trials reported major outcomes, we did not carry out subgroup analyses on different types of heart failure or different durations of treatment.

In addition, when compared with conventional treatment alone, Qili Qiangxin plus conventional treatment significantly improved cardiac function (RR 1.38, 95 % CI 1.29 to 1.48, I^2^ = 0 %; defined as an increase of two or more functional classes using NYHA) and QOL (MD −8.48 scores, 95 % CI −9.56 to −7.39, I^2^ = 24 %). Used as an adjunctive treatment, Qili Qiangxin was associated with a lower incidence of adverse events (RR = 0.56, 95 % CI 0.40 to 0.78, I^2^ = 19 %). Details are displayed in Table 2.

**Table 2:** Summary of findings of Qili Qiangxin plus conventional treatment compared to conventional treatment for heart failure

Outcomes	Illustrative comparative risks^a^ (95 % CI)		Relative effect (95 % CI)	No of Participants (studies)	Quality of the evidence (GRADE)	Comments
	Assumed risk	Corresponding risk				
	Conventional treatment	Qili Qiangxin plus Conventional treatment				
All-cause mortality or cardiovascular mortality	72 per 1000	38 per 1000 (20 to 77)	RR 0.53 (0.27 to 1.07)	539 (6 studies)	⊕ ⊕ ⊝⊝ low^b,c,d^	
Follow-up: 1 to 6 months						
Major cardiovascular events	598 per 1000	275 per 1000 (203 to 383)	RR 0.46 (0.34 to 0.64)	224 (3 studies)	⊕ ⊕ ⊕⊝ moderate^b,d^	
Follow-up: 3 to 6 months						
Hospitalizations due to heart failure	342 per 1000	167 per 1000 (118 to 223)	RR 0.49 (0.38 to 0.64)	669 (9 studies)	⊕ ⊕ ⊕⊝ moderate^b^	
Follow-up: 1 to 6 months						
cardiac function (defined as an increase of two or more functional classes using NYHA)	336 per 1000	464per 1000 (434 to 498)	RR 1.38 (1.29 to 1.48)	4603 (54 studies)	⊕ ⊕ ⊝⊝ low^b,e^	
Follow-up: 1 to 6 months						
Quality of life (QOL)		The mean QOL in the intervention groups was 8.48 lower (9.56 to 7.39 lower)		792 (10 studies)	⊕ ⊕ ⊝⊝ low^b,f^	
Follow-up: 1 to 12 months						
Adverse drug reaction (ADR)	35 per 1000	20 per 1000 (14 to 27)	RR 0.56 (0.40 to 0.78)	4846 (56 studies)	⊕ ⊕ ⊕⊝ moderate^b^	

Patient or population: patients with heart failure

Settings: in or out

Intervention: Qili Qiangxin plus Conventional treatment

Comparison: Conventional treatment

*CI* confidence interval, *RR* risk ratio

GRADE Working Group grades of evidence

High quality: Further research is very unlikely to change our confidence in the estimate of effect

Moderate quality: Further research is likely to have an important impact on our confidence in the estimate of effect and may change the estimate

Low quality: Further research is very likely to have an important impact on our confidence in the estimate of effect and is likely to change the estimate

Very low quality: We are very uncertain about the estimate

^a^The basis for the assumed risk (e.g. the median control group risk across studies) is provided in footnotes. The corresponding risk (and its 95 % confidence interval) is based on the assumed risk in the comparison group and the relative effect of the intervention (and its 95 % CI)

^b^The RCTs failed to reported the methods of randomized and concealment of allocation

^c^This outcome is a clinical endpoint

^d^Total number of events is less than 300

^e^Most of the trials have wide range of 95 % CI for effect estimate

^f^There was significant statistical heterogeneity among trials according to *I* ^*2*^ test

A total of 84 trials evaluated LVEF and were pooled with a random model. Pooled comparisons demonstrated that Qili Qiangxin plus conventional treatment had a statistically significant beneficial effect compared to conventional treatment alone in terms of LVEF (MD 5.87, 95 % CI 5.28 to 6.47). However, a significant degree of heterogeneity was detected (I^2^ = 91 %), and when take subgroup analysis on duration of treatment, large heterogeneity still existed. Tests for subgroup differences showed no significant difference in effect between the trials with different treatment duration. Meta-analysis of 24 trials demonstrated that Qili Qiangxin plus conventional treatment significantly reduced levels of NT-proBNP (MD −214.43 pg/ml, 95 % CI −269.42 to −159.45). The high level of heterogeneity (I^2^ = 96 %) in these trials however should be noted. Similarly, pooled comparison of 42 trials indicated that Qili Qiangxin plus conventional medicine significantly improved the 6MWD (MD 47.21 meters, 95 % CI 44.53 to 49.90) when compared with conventional medicine alone. Again, a considerable level of heterogeneity (I^2^ = 96 %) was observed.

Considering general low quality of included trials, we did not take sensitivity analyses based on study quality according to protocol.

#### Qili Qiangxin capsule plus conventional treatment versus placebo plus conventional treatment

**Fig. 3:**
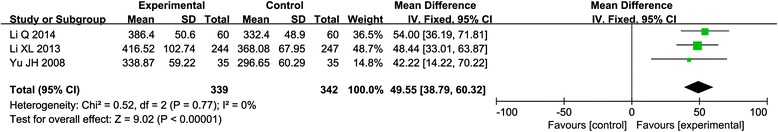
Forest plot of Qili Qiangxin plus conventional treatment versus placebo plus conventional treatment

Three trials were identified for this comparison. One multicenter double-blind trial (Li XL 2013) evaluated the composite cardiac events (CCEs) for 491 patients, and reported that CCE rate was 4.51 % in the Qili Qiangxin plus conventional treatment group, compared with 10.93 % in the placebo plus conventional treatment group (*p* < 0.05) [18]. This study also reported a favorable effect of Qili Qiangxin plus conventional treatment on the plasma NT-proBNP level, the NYHA functional classification and QOL by MLHFQ at 12 weeks (*p* < 0.001 for above outcomes). Meta-analysis of three trials (Fig. 3) showed statistically significant improvement of 6MWD in the Qili Qiangxin plus conventional medicine group compared to placebo plus conventional medicine (MD = 49.55 meters, 95 % CI 38.79 to 60.32, I^2^ = 0 %).

#### Qili Qiangxin capsule plus conventional treatment versus medications recommended in guidelines plus conventional treatment

Nine trials compared Qili Qiangxin capsule plus conventional treatment to supplementary medications recommended in clinical guidelines. The supplementary medications included thiazide diuretics, benazepril, captopril, metoprolol, irbesartan, trimetazidine, hydrochlorothiazide and digoxin.

Meta-analysis with a fixed model of four trials indicated no significant effect on cardiac function (RR = 1.26, 95 % CI 0.94 to 1.70, I^2^ = 4 %) for Qili Qiangxin plus conventional treatment when compared with supplementary medications plus conventional treatment. Further subgroup analyses on cardiac function according to medications found that Qili Qiangxin had no statistically significant differences compared to captopril (*n* = 2 trials, RR = 1.27, 95 % CI 0.89 to 1.82, I^2^ = 0 %), irbesartan plus trimetazidine (*n* = 1 trial, RR = 2.50, 95 % CI 0.83 to 7.49), or digoxin (*n* = 1 trial, RR = 0.85, 95 % CI 0.45 to 1.59).. Meta-analysis with a random model of four trials showed that Qili Qiangxin plus conventional treatment had significantly lower risk of adverse events (RR = 0.21, 95 % CI 0.06 to 0.74, I^2^ = 49 %). Two trials reported 6MWD and meta-analysis showed that Qili Qiangxin plus conventional treatment significantly improved 6MWD compared with supplementary medications (MD = 43.29 meters; 95 % CI =14.91 to 71.67, I^2^ = 58 %).

### Publication bias

**Fig. 4:**
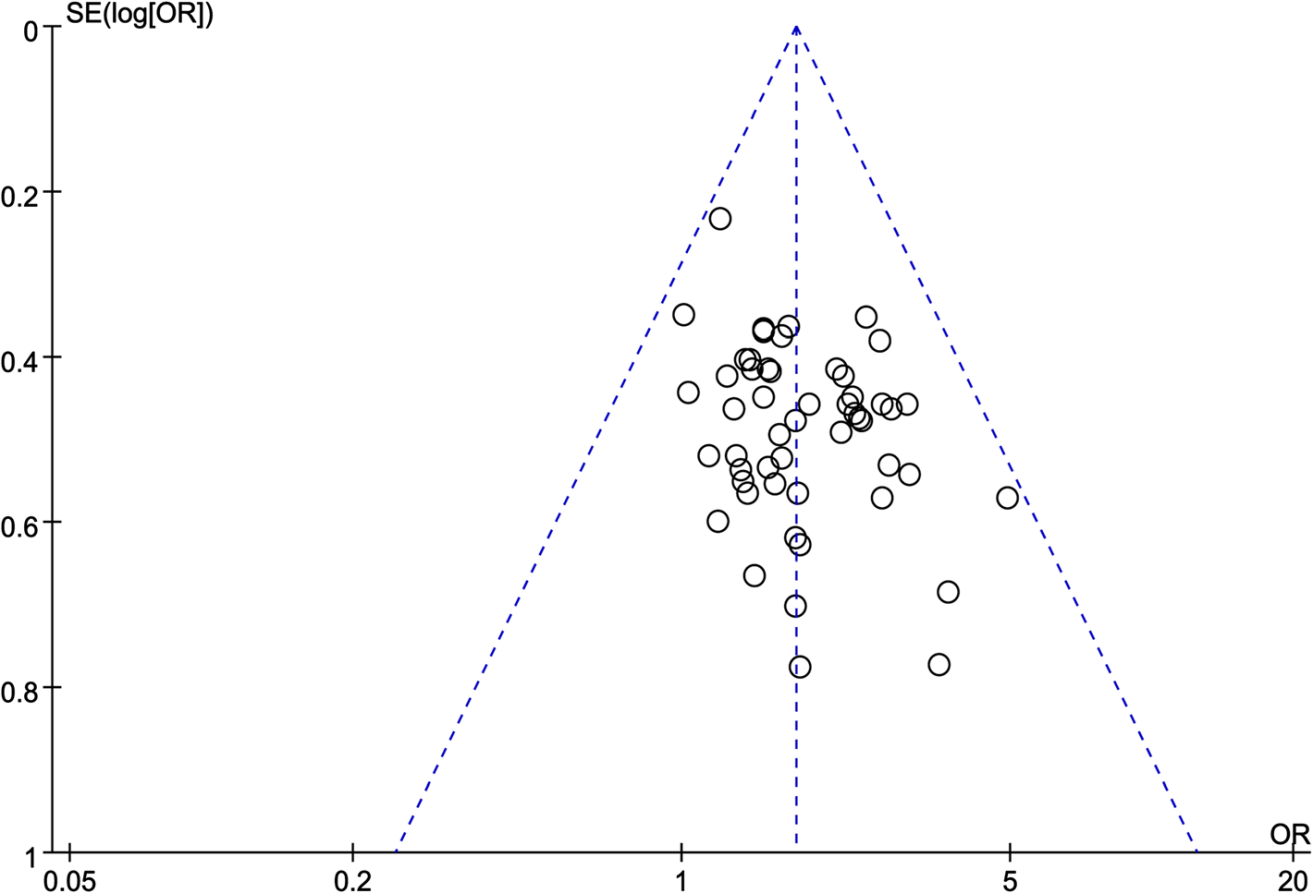
The funnel plot of publication bias

A funnel plot analysis was generated for 50 trials comparing Qili Qiangxin plus conventional treatment to conventional treatment alone for the outcome of NYHA levels. No asymmetry was observed, suggesting no publication bias (Fig. 4).

## Discussion

### Summary of findings

The findings of this review suggest that in patients with HF, Qili Qiangxin used as an adjunct to conventional treatment leads to a statistically significant reduction in major cardiovascular events and re-hospitalization due to HF when compared with conventional treatment alone. However, there appeared to be no additional effects of Qili Qiangxin in terms of mortality rate. Qili Qiangxin plus conventional treatment also appears to significant improvements in cardiac function measured by levels of NHYA and showed significant beneficial effects on NT-proBNP levels, QOL, LVEF and 6MWD. Pooled data indicated that Qili Qiangxin as an adjuvant treatment have clinical significance in improving exercise capacity as well as symptomatic status. Preliminary data suggests that Qili Qiangxin appears to be safe. However, it should be noted that the majority of included trials had low methodological quality and a high risk of bias. Any conclusions drawn from this review should therefore be interpreted with caution.

### Strength and limitations

This study presents the first comprehensive and rigorous review of Qili Qiangxin as a supplementary treatment for patients with HF. Previous criticisms have been made regarding the use of different terms to describe types of HF potentially being confusing [7]. It is strength of this review that we included all types of HF with different comorbidities in order to maximize the impact and clinical relevance of our findings.

There are a number of limitations to this review which need to be acknowledged. The majority of included studies were assessed as unclear risk of bias. This was largely owing to the lack of details, particularly in terms of random sequence generation, allocation concealment, blinding methods and availability of a protocol. Due to limited resources and time constraints we were not able to contact trial authors to request missing data and other information preventing us from being able to make a complete assessment regarding risk of bias. However, the sensitivity analysis indicated that the missing data not change the results in this review. Furthermore, the follow-up period in the included studies was no longer than six months, with the majority reporting a follow-up of three months or less. This makes it difficult to interpret the present evidence on mortality as an outcome, and in assessing the long-term effects and safety of administering Qili Qiangxin. We excluded 40 articles on the basis of no relevant outcomes reported. These articles did not report the outcomes we listed in protocol, instead, they chose composite outcome indicator. We excluded these articles as they failed to provide data of separate components. Considering all studies excluded for this reason reported positive results for the composite outcome, we believed the exclusion was unlikely to affect the results of our review.

### Previous studies

A recent editorial has suggested that Qili Qiangxin showed promising results. If Qili Qiangxin is shown to be safe and effective from further rigorous clinical trials research, this presents an interesting area of further work that may fundamentally challenge our current need to precisely understand the pharmacodynamics of all drug therapies [19]. Herbal medicines appear to operate through a variety of often poorly defined synergistic mechanisms involving multiple chemical components. In our study we found that Qili Qiangxin capsules have a positive effect on NT-proBNP levels. Previous studies showed that NT-proBNP level can be used as a prognostic marker for congestive heart failure as decreased NT-proBNP levels predicted reducing mortality in 10 years [20–22]. Levels of NT-proBNP clearly differ among various congenital heart lesions, and a higher level of NT-proBNP correlates with diastolic dysfunction parameters. NT-proBNP levels are related to exercise capacity and also increase with the more dysfunctional HF stages [23]. In our study, we included patients regardless of gender, age, etiology, ethnic group, severity, and course of diseases. Consequently the patients’ in different trials had different underlying diseases. All of these factors might explain the high heterogeneity in the meta-analysis of NT-proBNP. The high heterogeneity of LVEF and 6MWD might separately due to the different population baseline of LVEF and 6MWD in the included trials.

### Implications for future research

The results from this review suggest that further research is warranted in order to provide further evidence assessing the effects and safety of Qili Xiangxin as an adjuvant to conventional treatments for HF. We have a number of recommendations for future research. Various diagnostic criteria are used internationally for HF and future research should ideally use internationally recognized diagnostic criteria such as the ACC/AHA guidelines or ESC guidelines as part of their inclusion criteria. Further studies of Qili Qiangxin should also incorporate a minimum one year follow-up period in order that clinically important data on outcomes such as mortality and cardiac events can be provided. These were rarely reported amongst the RCTs we found in this review and further data in this area would be clinically meaningful to patients and providers. Furthermore, we found the reporting of clinical trial methods such as random sequence generation and allocation concealment inadequate and we recommend researchers report in full their trial methodology in future publications. Linked to this, none of the RCTs we included in this review provided trial protocols, and some did not provide all outcomes that had been described in the methods section. For transparency, we recommend that researchers prospectively register trials, publish trial protocols and cite the protocol or registration number in subsequent publications. This will enable future researchers and guideline developers to consider the evidence presented in light of what had been planned by the research team prior to trial commencement. Finally, we found few RCTs using placebo-control design in this review. Studies in future should adopt a double-blinded placebo-controlled design in order that further information regarding specific effects of Qili Qiangxin in HF can be provided.

## Conclusions

When compared with conventional treatment alone, Qili Qiangxin combined with conventional treatment demonstrated a significant effect in reducing cardiovascular events and re-hospitalization rate, though not in mortality. Qili Qiangxin appeared to be associated with an increased QOL and preliminary data suggested that it is safe. This data was drawn from low quality trials and the results of this review must therefore be interpreted with caution. Further rigorous research is warranted through large, prospective clinical trials in order to confirm these findings.

## Additional files

Additional file 1: PRISMA 2009 Checklist. (DOC 63 kb) Additional file 2: Supplemental reference list of all included study. (DOC 627 kb) Additional file 3: The risk of bias ratings and rationales for each included study. (DOC 201 kb) Additional file 4: Forest plots for all meta-analyses. (DOC 660 kb)
