# Supplementary material for: Clinical effects of a standardized Chinese herbal remedy, Qili Qiangxin, as an adjuvant treatment in heart failure: systematic review and meta-analysis
Source: BMC Complement Altern Med. 2016 Jul 11;16:201. doi: 10.1186/s12906-016-1174-1 (PMC4940829; doi:10.1186/s12906-016-1174-1)
Supplement: Additional file 2: — Supplemental reference list of all included study. (DOC 627 kb) [file 12906_2016_1174_MOESM2_ESM.doc]

**Supplemental reference list of all included study**

1. Bai LQ, Yu J, Jiang MH, Chen WQ, Yang RJ, Li YH. **Therapeutic effect of Qiliqiangxin capsule treating heart failure after delayed PCI for Acute Myocardial Infraction**. *World Chinese Medicine* 2013, **8**(6):686-8.
2. Cai RF. **Clinical** **observation of Qiliqiangxin capsule for treatment of chronic heart failure**. *The 9^th^ symposium on collateral disease theory solemnly*. Shanghai China; 2013: 3.
3. Cai YP, Zhu YW, Zhang AJ. **Effect of Qiliqiangxin capsule on serum galectin-3 in chronic heart failure**. *Chinese Journal of Integrative Medicine on Cardio/cerebrovascular Disease* 2013, **11**(7): 801-2.
4. Chen L, Chen WQ. **Observation on the therapeutic effect of Qiliqiangxin capsule in treating patients with chronic heart failure.** In: *The 5^th^ symposium on collateral disease theory solemnly*. Guangzhou China. 2009: 540-2
5. Chen TC, Sun FQ, Li M, Zhang L, Liu J. **Observation on the effect of Qiliqiangxin capsule on patients with refractory chronic heart failure.** *Chin J of Clinical Rational Drug Use* 2013, **6**(11): 35-36.
6. Chen WQ, Yu J, Bai LQ, Yang RJ, Wang F, Ma T, Jiang MH. **Clinical study on combination of Qiliqiangxin capsule with trimetazidine in patients with chronic heart failure.** *Chin J Diffic and Compl Cas* 2012, **11**(11): 830-2.
7. Chen XD. **The therapeutic effect of Qiliqiangxin capsules in patients with ischemic heart failure**. *Guide of China Medicine* 2013, **11**(10):25-26.
8. Cui LL, Zhang HY. **Clinical experience of Qiliqiangxin capsule in treatment of patients with chronic congestive heart failure***. Hebei J TCM* 2012, **34**(6): 815-8.
9. Dai JX. **Clinical effect of integrated traditional Chinese and Western medicine therapy for chronic congestive heart failure**. *Prevention and treatment of Cardiovascular Diseases* 2013, (7): 7-9.
10. Ding SY. **Clinical observation of Qiliqiangxin capsule combined with western medicine in the treatment of chronic heart failure**. *Chin J of Clinical Rational Drug Use* 2013, **6**(5):8-9.
11. Dong MX, Jiang JF, Li ZM. **Integrated Chinese and Western medicine therapy for patients with chronic heart failure**. *Practical Clinical Journal of Integrated Tradition 2*013, **13**(1): 12-13.
12. Du YK. **Clinical observation of Qiliqiangxin capsule in the treatment of chronic heart failure**.*Shanxi J of TCM* 2014, **30**(2): 14-15.
13. Duan JH, Yan GW, Liu X. **Effect observation of** **Qiliqiangxin capsule on patients of community with chronic heart failure.** *Chin J of Clinical Rational Drug Use* 2010, **3**(24): 6-7.
14. Fan J, FangF. **Clinical observation on Qiliqiangxin capsule combined western medicine treatment for improving quality of life in patients with chronic heart failure**. *Medical Information* 2013, **26**(2): 254-5.
15. Feng QT. **Qiliqiangxin capsule in the treatment of chronic congestive heart failure clinical observation and on the B-type natriuretic peptide intervention of clinical research**. *Hebei Medical University* 2013.
16. Fu JZ, Wei WH, Wang LM, Sun WY. **Qiliqiangxin capsule as an adjuvant therapy for chronic heart failure in aged patients.** *Chin J of Clinical Rational Drug Use* 2012, **5**(4):63-4.
17. Gao JB, Li YD, Yang SZ. **Qiliqiangxin capsule as a treatment for chronic heart failure.** *Chinese Journal of Experimental Traditional Medical Formulae* 2011, **17**(7): 233-4
18. Guan SY, Yang L. **Clinical study of Qiliqiangxin capsule in the adjuvant treatment of chronic heart failure with normal left ventricular ejection fraction**. *Chin J Diffic and Compl Cas* 2012, **11**(7):500-2.
19. Guo SL, Zhao SM, Liu L. **Clinical study of Qiliqiangxin capsules in the treatment of chronic heart failure**. *Guide of China Medicine* 2011, **9**(16):324-5.
20. Guo WB. **Clinical study on Qiliqiangxin capsules as a treatment for 35 patients with chronic heart failure**. *Guide of China Medicine* 2013, **11**(5):289-290.
21. Hu B. **Clinical study on Qiliqiangxin capsules as a treatment for patients with chronic heart failure**. *Clinical Medicine* 2013, **33**(12): 114-5
22. Huang B. **Effect of Qili Qiangxin Capsule on Cardiac Function and Levels of Brain Natriuretic Peptide in Patients with Chronic Systolic Heart Failure.** *Chinese Journal of Experimental Traditional Medical Formulae* 2010, **16**(16): 191-3.
23. Huang YQ. **Clinical study on Qiliqiangxin capsules for patients with chronic heart failure**. *China Health Industry* 2012, (20):75-6.
24. Huang Z, Zhang XL, Zhang C, You YY, Zhang CH, Su H, Yan J. **The inhibition of Qiliqiangxin capsule on ventricular remodeling in patients with CHF**. *The 10^th^ symposium on collateral disease theory solemnly*. Beijing China;2014, 595-7
25. Jin Y, Cui SL, Jin P, Liu DL, Lu ZT, Li L. **Effects of Qiliqiangxin capsule for 50 cases of ischemic cardiomyopathy**. *Chinese Journal of Coal Industry Medicine* 2012, **15**(10): 1490-2.
26. Jing GJ. **Effects of Qiliqiangxin capsule for 30 cases of** **systolic heart failure**. *Hebei J TCM* 2009, **31**(7): 1060-1.
27. Kuang JB, Huang RJ, Pan AQ, Yang Y, Cen YH, et al. **Regulation of qiliqiangxin capsule on abnormal activation of nerve and endocrine in patient with chronic heart failure**. *Chinese Journal of New Clinical Medicine* 2008, **1**(1): 19-23.
28. Li DW. **Qiliqiangxin capsule in treatment of congestive heart failure**. *Chinese Journal of Practical Medicine* 2013, **40**(1): 37-9.
29. Li GM, Zhang HQ, Zhang CY. **Clinical Observation of Qiliqiangxin Capsule Combined with Western Medicine in the Treatment of Chronic Heart Failure.** *Research of Integrated Traditional Chinese and Western Medicine* 2011, **3**(6):285-7.
30. Li P, **Efficacy observation of Qiliqiangxin Capsule for treating chronic heart failure by 6-min walk test**. *Modern Medicine and Health* 2011, **27**(16):2430-1.
31. Li RY, Fan YK, Li XY, Qi XY. **Clinical observation on the treatment of chronic heart failure with Qiliqiangxin capsule**. *Int J Trad Chin Med* 2010, **32**(5): 409-10.
32. Li SQ, Zhang FX**.** **The clinical research of Qiliqiangxin capsule on CHF.** *The 10^th^ symposium on collateral disease theory solemnly*. Beijing China;2014, 611-2.
33. Li SZ. **The clinical research of Qiliqiangxin capsule on congestive heart failure of coronary artery disease.** *Medical and Pharmaceutical World* 2009, **11**(9):509-510.
34. Li T, Li Z. **The clinical research of Qiliqiangxin capsule on CHF**. *Chinese J Ind Med* 2010, **23**(6):477
35. Li WY, Lu TQ, Yu J, Sun T, Wang Q, Cai ZW. **The effect of Chinese patent medicine intervention on heart failure.** *Chinese Journal of Cardiovascular Research* 2013. **11**(5):357-60
36. Li YH, Zhou R, Yue LY. **Clinical efficiency observation on Qiliqiangxin capsule in treating chronic heart failure with special complications.** *The 9^th^ symposium on collateral disease theory solemnly*. Shanghai China; 2013:269-71.
37. Li YX, Yuan GQ, Jia ZH. **The clinical research of Qiliqiangxin capsule on diastolic heart failure.** *Chinese Journal of Basic Medicine in Traditional Chinese Medicine* 2012, **18**(3): 289-91.
38. Li YX, Jia ZH, Zhang XY. **Effect of Qiliqiangxin capsules on diastolic heart function in patients with chronic diastolic heart failure.** *Clin J Diffic and Compl Cas* 2013, **12**(4):261-3.
39. Lin JH, Wu PB, Cai WY, Xu XB, Zhuang MR, Li CL. **C****linical observation on Qiliqiangxin capsules in treating chronic heart failure.** *Chinese Medicine Modern Distance Education of China* 2008, **6**(3):254-5.
40. Lin ZJ. **Integrated Chinese and western medicines in treatment of heart failure**. *Journal of China traditional Chinese medicine information* 2010, **2**(32):218
41. Liu HL, An H. **Integrated Chinese and western medicines in treatment for aged patients with heart failure**. *Clin J Diffic and Compl Cas* 2008, **7**(11):671-2.
42. Liu J, Liu G, Wang TJ, Liu JT. C**linical observation on Qiliqiangxin capsules in treating chronic heart failure.** *Clinical Medicine* 2008,**28**(11):120-1.
43. Liu LX, Wu ZH, Zhu XG. **Therapeutic effect of Qiliqiangxin capsule on the treatment of chronic renal insufficiency heart failure patients**. *Clin J Diffic and Compl Cas* 2014, **13**(4):342-4.
44. Liu SJ. **Therapeutic effect of Qiliqiangxin capsule on the treatment of chronic heart failure**. *The 5^th^ symposium on collateral disease theory solemnly*. Guangzhou China. 2009:543-5
45. Liu T, Li L, Guo ZQ, Gao B, Zeng XH, Gu Y. **Effects of Qiliqiangxin capsule on patients with chronic pulmonary heart disease and heart failure.** *Clin J Diffic and Compl Cas* 2013, **12**(2)：85-7
46. Liu TR, Tang JG. **Clinical effectiveness of Qiliqiangxin capsules for chronic heart failure.** *Journal of Bengbu Medical College* 2010, **35**(12): 1282-3.
47. Liu WJ, Shi YP, Yang HY, Liao XC. **Clinical effectiveness of Qiliqiangxin capsules for quality of life on patients with chronic heart failure.** *Clin J Diffic and Compl Cas* 2007, **6**(11): 675-6.
48. Liu XC. **Clinical effectiveness of Qiliqiangxin capsules for chronic heart failure.** *The 4^th^ symposium on collateral disease theory solemnly*. Nanjing China. 2008:361-2.
49. Liu XC, Zhang ZX, Chen XL, Li SJ, Wang HH, Zhang XY. **Clinical effectiveness of Qiliqiangxin capsules for chronic heart failure.** *Chinese Journal of Integrative Medicine on Cardio/cerebrovascular Disease* 2011, **9**(8):905-6.
50. Liu XG. **The clinical observation of Qiliqiangxin capsule in the treatment of chronic heart failure**. *Hubei University of Chinese Medicine* 2013.
51. Liu XM, Song YP, Gu MF. **Clinical effectiveness of Qiliqiangxin capsules for chronic heart failure***. Chin J of Clinical Rational Drug Use* 2010, **3**(18): 56-37.
52. Liu XM, Quan XH, Ma CY, Ma X, Li YJ, Zhu JY, Lu YQ. **Clinical observation of Qiliqiangxin capsule for treatment of elderly patients with chronic heart failure**. *Chinese Journal of Difficult and Complicated Cases* 2013, **12**(4): 270-2.
53. Liu YJ. **Clinical observation of Qiliqiangxin capsule for treatment of elderly patients with heart failure.** *Medical Frontier* 2012, **2**(19):192-3.
54. Long F, Jiang S. **Therapeutic Effect of Qiliqiangxin Capsule on 110 Cases of Chronic Heart Failure.** *Liaoning Journal of Traditional Chinese Medicine* 2009, **36**(4):583-3.
55. Lu JP, Chen L, Li X, Yang RY. **Efficiency and security of Qiliqiangxin capsule in treatment of chronic heart failure.** *Journal of New Medicine* 2012, **22**(6):420-2.
56. Luo Q, Ye RL, Song YH. **Efficiency of** **Qiliqiangxin capsule in treatment of chronic heart failure**. *Journal of New Chinese Medicine* 2013, **45**(4):12-5.
57. Ma AP, Jin JY. **Qiliqiangxin capsule in treatment of chronic heart failure**. *Guide of China Medicine* 2013, **11**(24):301-2
58. Ma L, Jin HL. **Therapeutic** **Effect of Qiliqiangxin Capsule on patients with Chronic Heart Failure.** *Journal of Shanxi College of Traditional Chinese Medicine* 2010, **11**(4): 30-31.
59. Miao S, Zhang ZJ. **Influence of Qiliqiangxin capsule on cardiac function and plasma NT-proBNP concentration in elder patients with heart failure.** *The 9^th^ symposium on collateral disease theory solemnly*. Shanghai China; 2013: 295-7.
60. Niu LY, Zhang LN, Zhao XJ. **The effect of Qiliqiangxin capsule to elder patients suffered from chronic systolic heart failure and the level of serum BNP.** *Chinese Journal of Difficult and Complicated Cases* 2012, **11**(10): 741-3.
61. Pang XM, Liu YH, Bai BQ. **Qiliqiangxin capsule combined digoxin therapy for patients with chronic heart failure.** *The 4^th^ symposium on collateral disease theory solemnly*. Nanjing China. 2008:366.
62. Qiu X. **Effect of Qiliqiangxin Capsule on patients with Chronic Heart Failure**. *Guide of Ghina Medicine* 2013, **11**(36): 212-3.
63. Rao LZ, Xiong H, Dai WQ. **Influence of Qiliqiangxin capsule on patients with heart failure.** *Chinese Journal of Difficult and Complicated Cases* 2012, **11**(5): 374-5
64. Shi CP, Zhang XS, Rao SG, Wang AJ, Liu AH. **Influence of Qiliqiangxin capsule on patients with heart failure**. Journal of New Chinese Medicine 2013, **45**(6): 14-5.
65. Su HM. **Effect of Qiliqiangxin Capsule on patients with Chronic Heart Failure.** *Chinese Journal of Integrative Medicine on Cardio/cerebrovascular Disease* 2007, **5**(10): 917-8.
66. Su LJ, Wang JZ, Fang ZH. **Therapeutic** **effects of Qiliqiangxin capsule on chronic heart failure**. *Chinese Journal of Cardiovascular Rehabilitation Medicine* 2012, **21**(2): 199-201.
67. Su RY. **Effect of Qiliqiangxin Capsule on elderly patients with Chronic Heart Failure.** *National Medical Frontiers of China* 2013, **8**(14): 21-3.
68. Sun LP, Zhang LC. **Therapeutic effect of Qiliqiangxin capsule on the patients with chronic congestive heart failure**. *Chinese Journal of Difficult and Complicated Cases* 2007, **6**(2):71-2.
69. Tang SY, Wang DX, Yan FF, Liu Y, Fang F, Liu XF, et al. **Therapeutic effect of Qiliqiangxin capsule on the patients with chronic congestive heart failure**. *Chinese Journal of Gerontology* 2013, **33**(17): 4106-8.
70. Tao X, Gao YH, Li WQ, Dong XY, Luo Q, Qin WJ, et al. **Effects of Qiliqiangxin capsule on chronic heart failure**. *Chinese journal of ethnomedicine and ethnopharmacy* 2011, (13): 91-3
71. Tian Y, Li YX, Ren JX, Han SL, Liu XF, Gao XD. **Observation effects of Qiliqiangxin capsule for diastolic heart failure.** *The Chinese Journal of Clinical Pharmacology* 2011, **27**(9): 666-8.
72. Wang N. **Effect of Qiliqiangxin Capsule on patients with Chronic Heart Failure**. Shaanxi Traditional Chinese Medicine 2014, **35**(2): 131-2.
73. Wang Q, Wei B, Fang XM. **Effect of Qiliqiangxin Capsule on patients with Chronic Heart Failure.** Chinese Community Doctors 2012.
74. Wang SZ, Liu P, Zhou Y, Cui FS, Li CE, Zhang QJ, Yang RC. **Effect of Qiliqiangxin Capsule on patients with cardiac insufficiency.** *Chinese Journal of Difficult and Complicated Cases* 2012, **11**(2): 125-6.
75. Wang YY. **Effect of Qiliqiangxin capsule on patients with chronic congestive heart failure.** *World Health Digest* 2013, (47): 108.
76. Wei XB, Mai M. **The clinical effect of Qiliqiangxin capsules in treatment of elderly patients with chronic heart failure.** *China Modern Doctor* 2013, **51**(35): 82-4.
77. Wen Y, Lan WQ, Liu RQ. **The clinical effect of Qiliqiangxin capsules in treatment of 45 patients with chronic heart failure.** *Journal of Community Medicine* 2012, **10**(21): 29-30
78. Xiong SQ, Zhang XM, Wang TT, Chen Y, Jin QL, Gao YY. **Curative effect observation of Qiliqiangxin capsule in the treatment of chronic heart failure.** *The 10^th^ symposium on collateral disease theory solemnly*. Beijing China; 2014, 588-90.
79. Xu GS, Hu Q. **Curative effect observation of Qiliqiangxin capsule in the treatment of cardiac insufficiency.** Chin J of Clinical Rational Drug Use 2014, **7**(7): 50-51.
80. Xue L, Sun YH. **Effects of Qili Qiangxin capsules on inflammatory cytokine levels in patients with chronic heart failure.** *Prevention and treatment of Cardiovascular Diseases* 2014, (5): 64-67.
81. Xue LX, Feng L. **The clinical effect of Qiliqiangxin capsules in treatment of 45 patients with chronic congestive heart failure.** *Chinese Journal of Integrative Medicine on Cardio/cerebrovascular Disease* 2008, **6**(5): 584-5.
82. Yan KL, Xiao B, Ou TH. **Therapeutic effects of Qiliqiangxin capsule on chronic heart failure patients with renal insufficiency**. *Chinese Journal of Difficult and Complicated Cases* 2012, **11**(12): 906-7.
83. Yan DC, Dong XY. **The clinical effect of Qiliqiangxin capsules in treatment of patients with heart failure.** *Medical Frontier* 2014, (1): 126-7
84. Yang F, Lai SY. **The clinical effect of Qiliqiangxin capsules in treatment of patients with chronic heart failure.** *Chinese Journal of Integrative Medicine* 2007, **27**(11): 1041-2.
85. Yang HT. **The clinical effect of Qiliqiangxin capsules in treatment of patients with chronic heart failure**. *Hunan Journal of Traditional Chinese Medicine* 2012, **28**(5): 21-2.
86. Yang HT. **The clinical effect of Qiliqiangxin capsules in treatment of patients with chronic heart failure**. *Henan Traditional Chinese Medicine* 2013, **33**(2): 282-283.
87. Yang J, Zhao JS, Wei YL. **Clinical Research of Cordialina Oral Liquid in the Treatment of Chronic Cardiac Failure.** *China Journal of Chinese Medicine* 2013, **28**(186): 1721-3.
88. Yang W, Zhao FC, Yang YY, Wang YH. **Effect of Qili Qiangxin Capsule on Cardiac Function and Plasma Antidiuretic Hormone in Patients with Chronic Heart Failure**. *Chinese Journal of Experimental Traditional Medical Formulae* 2012, **18**(3): 200-2.
89. Yao L, Li YX, Liu LN, Wang Y, Hu YM. **The therapeutic effects of Qiliqiangxin capsule in 50 patients with chronic heart failure.** *Chinese Journal of Difficult and Complicated Cases* 2011, **10**(12):886-8.
90. Ye RS, Xiong YQ, Yang XL. **Clinical observation of Qiliqiangxin capsule on the treatment of elderly patients with chronic heart failure**. *The 9^th^ symposium on collateral disease theory solemnly*. Shanghai China; 2013:298-300.
91. Ye S, Cao Q. **Clinical observation of Qiliqiangxin capsule on the treatment of patients with chronic heart failure**. *Clinical Journal of Microcirculation* 2012, **22**(2): 60-1.
92. Yin ZL, Deng P. **Clinical observation of Qiliqiangxin capsule on the treatment of patients with chronic heart failure**. *Journal of Clinical Research.* 2009, **26**(4): 720-2.B
93. Ying M, E ED. Hai L. **Clinical observation of Qiliqiangxin capsule on the treatment of patients with chronic heart failure**. *People’s Military Surgeon* 2013, **56**(7): 799-805.
94. Yuan JK. **Effects of Qiliqiangxin capsule on cardiac function and levels of serum brain natriuretic peptide in dilated cardiomyopathy patients with heart failure**. *Modern Medicine Journal of China* 2012, 14(11): 40-2.
95. Zhang CA. **Observation on the efficacy of the Qiliqiangxin capsule on cardiac function and mental state of patients with chronic heart failure.** *The 9^th^ symposium on collateral disease theory solemnly*. Shanghai China; 2013: 272-4.
96. Zhang H. **The therapeutic effects of Qiliqiangxin capsule on 71 patients with chronic heart failure.** *Journal of China Traditional Chinese Medicine Information* 2011, 3(11): 89.
97. Zhang WL. **The therapeutic effects of Qiliqiangxin capsule in patients with chronic heart failure.** *Inner Mongol Journal of Traditional Chinese Medicine* 2013, 32(18).
98. Zhang XX. **Observation on the efficacy of the Qiliqiangxin capsule on cardiac insufficiency.** *Inner Mongol Journal of Traditional Chinese Medicine* 2010, **29**(12):14-5.
99. Zhao JS, Yang J, Wei YL, Xiao XQ, Yi WG, Liu H. **Observation on the efficacy of the Cordialina Oral Liquid on heart failure.** *Henan Traditional Chinese Medicine* 2014, 34(2): 246-8.
100. Zhao MJ, Zheng XL. **Observation on the efficacy of the Qiliqiangxin capsule on patients with heart failure.** *The 5^th^ symposium on collateral disease theory solemnly*. Guangzhou China. 2009: 548-9
101. Zheng MJ, Liu DM. **Efficiency observation on Qiliqiangxin capsule in assistant treatment of heart failure due to dilated cardiomyopathy.** *The 8^th^ symposium on collateral disease theory solemnly*. China. 2012:284-6
102. Zheng JJ, Lan WQ, Ma JD, Liu RQ. **Observation on the efficacy of the Qiliqiangxin capsule on patients with heart failure.** *Journal of Psychologist* 2012, (8):273-4.
103. Zheng LW, Liu C. **Effect of Qiliqiangxin capsule on heart function and NT-proBNP in elderly patients with chronic heart failure**. *The 9^th^ symposium on collateral disease theory solemnly*. Shanghai China; 2013: 287-9.
104. Zheng WH. **Observation on the efficacy of the Qiliqiangxin capsule on patients with heart failure.** *Journal of Emergency in Traditional Chinese Medicine* 2014, **23**(3): 526-7.
105. Zhou FZ. Clinical study of Qiliqiangxin capsule combined with perindopril in treating congestive heart failure. China Medicine 2011, 6(6): 650-2.
106. Zhou Y, Zheng CH. **Clinical Observation of Qiliqiangxin Capsule in the Treatment of Diabetic Patients with Ventricular Dysfunction.** *Hubei Journal of Traditional Chinese Medicine* 2013, **35**(12): 6-7.
107. Zhu HG, Liu WH. **Observation on the efficacy of the Qiliqiangxin capsule on patients with heart failure.** *Guide of China Medicine* 2012, 10(36): 288-9.
108. Zhuo JY, Xu Y, Nie Q, Yin YJ. **Effects of Qiliqiangxin capsule on cardiac function in patients with chronic heart failure and article fibrillation**. Med J West China 2013, 25(3): 392-4.
109. Chen XH, Sun Q, Li L, Liang JY, Meng WG, Liu JB. **Effect of Qiliqiangxin capsules on neurohumoral factors,left ventricular remodeling and heart function in the patients with ischemic heart disease with heart failure.** *Modern Journal of Integrated Traditional Chinese and Western Medicine* 2014, **23**(25): 2760-3.
110. Guo P, Liu RH. **The clinical evaluation on patients suffered from ischemic heart failure of long-term administration with Qiliqiangxin Capsule**. *The 10^th^ symposium on collateral disease theory solemnly*. Beijing China, 2014, 627-9.
111. Ma RX, Liu YH, Wang N, Zhang X. **Effect of Qiliqiangxin capsules on patients with chronic heart failure.** *Hebei Medical Journal* 2014, **36**(23):3602-3
112. Shen XR. **Effect of Qiliqiangxin capsules on patients with chronic cardiac dysfunction.***Shanxi Med J* 2014, **43**(23): 2803-4.
113. Wu GL, Chen AL. **Clinical Observation of Chronic Congestive Heart Failure Treated by Qiliqiangxin Capsule for 52 Cases.** *Chinese Medicine Modern Distance Education of China* 2015, **13**(3): 23-4.
114. Wu SP, Chang LP, Yuan GQ, Li YX. **Clinical observation of Qiliqiangxin capsules improved vascular endothelial function on patients with chronic systolic heart failure.** *Chinese Journal of Difficult and Complicated Cases* 2014, **13**(8): 827-30.
115. Wu X. **Clinical Observation of Qiliqiangxin Capsule in treating Coronary Heart Disease with Chronic Heart Failure of Qi and Yang Deficiency, Blood Stasis and Water Stagnation Syndrome.** *Hunan University of Chinese Medicine* 2014.
116. Zhai N, Zhang YJ, Wang MY, Xu J. **Clinical effect observation of Qiliqiangxin capsules in treatment of chronic heart failure.** *China Modern Medicine* 2015, **22**(4): 72-4.
117. Zhang J. **Therapeutic Effect of Qili Qiangxin Capsule on the Patients with Chronic Heart Failure**. *Journal of Liaoning University of TCM* 2015, **17**(3): 155-7.
118. Zhang R, Zhang L, Zhang JW, Tian L, Li XX. **The effect of Qiliqiangxin capsule on heart function in the patients with chronic renal failure combined with chronic congestive heart failure.** *Chinese Journal of Difficult and Complicated Cases* 2014, **13**(12): 1279-82.
119. Li X, Zhang J, Huang J, et al. **A multicenter, randomized, double-blind, parallel-group, placebo-controlled study of the effects of qiliqiangxin capsules in patients with chronic heart failure.** *J Am Coll Cardiol* 2013, **62**(12): 1065-1072.
120. Yu JH. **Significance of plasma hs-CRP level in diastolic heart failure and intervention effect of Qiliqiangxin Capsule.** *Shandong Traditional Chinese Medicine University* 2008.
121. Li Q, Guo ZB, Li QM, Guo SH. **Effects of Qili Qiangxin capsule on serum concentration of adiponectin and heart function in patients with coronary heart disease combined with congestive heart failure**. *Chinese Journal of Pathophysiology* 2014, **30**(6): 1119-22.
122. Ding LB, Kong J, Du YJ. **Clinical observation of diabetic cardiomyopathy patients with heart failure treated by combination of Qiliqiangxin capsule and western medicine.** *Hebei J TCM* 2010, **32**(9): 1390-2.
123. Gu XM, Zhou RJ, Tong X, He XL. **Clinical effect observation of Qiliqiangxin capsules in treatment of chronic heart failure.** *The 5^th^ symposium on collateral disease theory solemnly*. Guangzhou China. 2009: 548-9.
124. Gu XM, Zhou XM, Tong X, Li GQ, Wu SB, Yin WH. **The effects of Qiliqiangxin capsule on symptoms and heart function of 35 patients with chronic heart failure.** *The 9^th^ symposium on collateral disease theory solemnly*. Shanghai China; 2013:275-7.
125. Gu YY. **The effects of Qiliqiangxin capsules in patients with chronic heart failure.** *Guangming Journal of Chinese Medicine,* 2012, **27**(5): 932-3.
126. Guan SY, Yang L, Yang F. **Clinical study of Qiliqiangxin capsule in the treatment of 38 cases of chronic congestive heart failure.** *Chin J Diffic and Compl Cas* 2013, **12**(4): 267-9.
127. Li LC, Tian SY, Fan JS, Wang YX. **The effects of Qiliqiangxin capsule for patients with chronic heart failure.** *Shaanxi Journal of Traditional Chinese Medicine* 2013, **34**(7): 872-3.
128. Ma FF, Lu FY, Zhao ZJ, Wang QL, Feng SW. **Effects of Qili Qiangxin capsule on endothelial function in patients with chronic heart failure.** *Shanghai Journal of Traditional Chinese Medicine* 2008, **42**(10): 18-20.
129. Ma FF, Lu FY, Xiao WG, Wang QL, Feng SW. **The effects of Qiliqiangxin capsule for patients with chronic heart failure.** *Chin J Diffic and Compl Cas* 2008, **7**(9):543-4.
130. Ma FF, Lu FY, Feng SW, Li YX, Xiao WG. **Influence of Qiliqiangxin capsule on vascular endothelial function and heart function in patients with chronic heart failure.** Modern Journal of Integrated Traditional Chinese and Western Medicine 2008, 17 (17): 2602-3.
131. Shen R. **The effects of Qiliqiangxin capsule for patients with chronic heart failure.** *Clinical* Medicine 2010, 30(3): 102-103.
