# Supplementary material for: Clinical effects of a standardized Chinese herbal remedy, Qili Qiangxin, as an adjuvant treatment in heart failure: systematic review and meta-analysis
Source: BMC Complement Altern Med. 2016 Jul 11;16:201. doi: 10.1186/s12906-016-1174-1 (PMC4940829; doi:10.1186/s12906-016-1174-1)
Supplement: Additional file 3: — The risk of bias ratings and rationales for each included study. (DOC 201 kb) [file 12906_2016_1174_MOESM3_ESM.doc]

**Additional file 3. The risk of bias ratings and rationales for each included study**

| **ID** | **Random sequence generation (selection bias)** | **Allocation concealment (selection bias)** | **Blinding of participants and personnel (performance bias)** | **Blinding of outcome assessment (detection bias)** | **Incomplete outcome data (attrition bias)** | **Selective reporting (reporting bias)** | **Other bias** | **Support for judgments** |
| --- | --- | --- | --- | --- | --- | --- | --- | --- |
| Bai LQ 2013 | Unclear^1^ | Unclear^2^ | Unclear^2^ | Unclear^2^ | Unclear^2^ | Low^3^ | Unclear^4^ | 1.NR 2.NM 3. RO 4. NB |
| Cai RF 2013 | Unclear^1^ | Unclear^2^ | Unclear^2^ | Unclear^2^ | Unclear^2^ | Low^3^ | Unclear^4^ | 1.NR 2.NM 3. RO 4. NB |
| Cai YP 2013 | Low^1^ | Unclear^2^ | Unclear^2^ | Unclear^2^ | Unclear^2^ | High^3^ | Unclear^4^ | 1.Using random number table generate the random sequence 2.NM 3. Not reported all outcomes listed in the methods (blood glucose, blood lipids) 4. NB |
| Chen L 2009 | Unclear^1^ | Unclear^2^ | Unclear^2^ | Unclear^2^ | Unclear^2^ | Low^3^ | Unclear^4^ | 1.NR 2.NM 3. RO 4. NB |
| Chen TC 2013 | Unclear^1^ | Unclear^2^ | Unclear^2^ | Unclear^2^ | Unclear^2^ | High^3^ | Unclear^4^ | 1.NR 2.NM 3. Not reported all outcomes listed in the methods (cardiac index, peripheral vascular resistance) 4. NB |
| Chen WQ 2012 | Unclear^1^ | Unclear^2^ | Unclear^2^ | Unclear^2^ | Low^3^ | Low^4^ | Unclear^5^ | 1.NR 2.NM 3. The author committed in the article that no person drop out, fall off or loss of follow up in text 4. RO 5. NB |
| Chen XH 2014 | Unclear^1^ | Unclear^2^ | Unclear^2^ | Unclear^2^ | Unclear^2^ | Low^3^ | Unclear^4^ | 1.NR 2.NM 3. RO 4. NB |
| Cheng XD 2013 | Unclear^1^ | Unclear^2^ | Unclear^2^ | Unclear^2^ | Unclear^2^ | Low^3^ | Unclear^4^ | 1.NR 2.NM 3. RO 4. NB |
| Cui LL 2012 | Unclear^1^ | Unclear^2^ | Unclear^2^ | Unclear^2^ | Unclear^2^ | Low^3^ | Unclear^4^ | 1.NR 2.NM 3. RO 4. NB |
| Dai JX 2013 | Unclear^1^ | Unclear^2^ | Unclear^2^ | Unclear^2^ | Unclear^2^ | High^3^ | Unclear^4^ | 1.NR 2.NM 3. Not reported all outcomes listed in the methods 4. NB |
| Ding LB 2010 | Unclear^1^ | Unclear^2^ | Unclear^2^ | Unclear^2^ | Unclear^2^ | Low^3^ | Unclear^4^ | 1.NR 2.NM 3. RO 4. NB |
| Ding SY 2013 | Unclear^1^ | Unclear^2^ | Unclear^2^ | Unclear^2^ | Unclear^2^ | Low^3^ | Unclear^4^ | 1.NR 2.NM 3. RO 4. NB |
| Dong MX 2013 | Unclear^1^ | Unclear^2^ | Unclear^2^ | Unclear^2^ | Unclear^2^ | Low^3^ | Unclear^4^ | 1.NR 2.NM 3. RO 4. NB |
| Du YK 2014 | Low^1^ | Unclear^2^ | Unclear^2^ | Unclear^2^ | Unclear^2^ | Low^3^ | Unclear^4^ | 1.Using sortition randomization method 2.NM 3. RO 4. NB |
| Duan JH 2010 | Unclear^1^ | Unclear^2^ | Unclear^2^ | Unclear^2^ | Unclear^2^ | Low^3^ | Unclear^4^ | 1.NR 2.NM 3. RO 4. NB |
| Fan J 2013a | Unclear^1^ | Unclear^2^ | Unclear^2^ | Unclear^2^ | Unclear^2^ | High^3^ | Unclear^4^ | 1.NR 2.NM 3. Not reported all outcomes listed in the methods (BNP, LVEF) 4. NB |
| Feng QT 2013 | Low^1^ | Unclear^2^ | Unclear^2^ | Unclear^2^ | Unclear^2^ | Low^3^ | Unclear^4^ | 1.Using random number table generate the random sequence 2.NM 3. RO 4. NB |
| Fu JZ 2012 | Unclear^1^ | Unclear^2^ | Unclear^2^ | Unclear^2^ | Low^3^ | Low^4^ | Unclear^5^ | 1.NR 2.NM 3.The author committed in the article that no person drop out, fall off or loss of follow up in text 4. RO 5. NB |
| Gao JB 2011 | Unclear^1^ | Unclear^2^ | Unclear^2^ | Unclear^2^ | Unclear^2^ | Low^3^ | High^4^ | 1.NR 2.NM 3.RO 4.Not reported comparability of baseline data and sample size calculation. |
| Gu XM 2009 | Unclear^1^ | Unclear^2^ | Unclear^2^ | Unclear^2^ | Unclear^2^ | Low^3^ | Unclear^4^ | 1.NR 2.NM 3. RO 4. NB |
| Gu XM 2013 | Unclear^1^ | Unclear^2^ | Unclear^2^ | Unclear^2^ | Unclear^2^ | Low^3^ | Unclear^4^ | 1.NR 2.NM 3. RO 4. NB |
| Gu YY 2012 | Unclear^1^ | Unclear^2^ | Unclear^2^ | Unclear^2^ | Unclear^2^ | Low^3^ | High^4^ | 1.NR 2.NM 3. RO 4.Not reported comparability of baseline data and sample size calculation. |
| Guan SY 2012 | Unclear^1^ | Unclear^2^ | Unclear^2^ | Unclear^2^ | Unclear^2^ | Low^3^ | Unclear^4^ | 1.NR 2.NM 3. RO 4. NB |
| Guan SY 2013 | Unclear^1^ | Unclear^2^ | Unclear^2^ | Unclear^2^ | Unclear^2^ | Low^3^ | Unclear^4^ | 1.NR 2.NM 3. RO 4. NB |
| Guo P 2014 | Unclear^1^ | Unclear^2^ | Unclear^2^ | Unclear^2^ | Low^3^ | Low^4^ | Unclear^5^ | 1.NR 2.NM 3. Authors reported 5 (5%) participants loss of follow-up and droped out. 4. RO 5. NB |
| Guo SL 2011 | Unclear^1^ | Unclear^2^ | Unclear^2^ | Unclear^2^ | Unclear^2^ | High^3^ | Unclear^4^ | 1.NR 2.NM 3. Not reported all outcomes listed in the methods (LVDs) 4. NB |
| Guo WB 2013 | Unclear^1^ | Unclear^2^ | Unclear^3^ | Unclear^2^ | High^4^ | Low^5^ | Unclear^6^ | 1.NR 2.NM 3. Used single blind method, but failed to mention who was blinded 4.Six cases less than the number at baseline without providing further explanation 5. RO 6. NB |
| Hu B 2013 | Unclear^1^ | Unclear^2^ | Unclear^2^ | Unclear^2^ | Unclear^2^ | High^3^ | Unclear^4^ | 1.NR 2.NM 3. Not reported all outcomes listed in the methods (blood lipid, blood glucose, liver function, renal function, electrolyte) 4. NB |
| Huang B 2010 | Low^1^ | Unclear^2^ | Unclear^2^ | Unclear^2^ | Unclear^2^ | High^3^ | Unclear^4^ | 1.Using random number table generate the random sequence 2.NM 3. Not reported all outcomes listed in the methods (Traditional Chinese Medicine Syndrome Score) 4. NB |
| Huang YQ 2012 | Unclear^1^ | Unclear^2^ | Unclear^2^ | Unclear^2^ | Unclear^2^ | Low^3^ | Unclear^4^ | 1.NR 2.NM 3. RO 4. NB |
| Huang Z 2014 | Unclear^1^ | Unclear^2^ | Unclear^2^ | Unclear^2^ | Unclear^2^ | Low^3^ | Unclear^4^ | 1.NR 2.NM 3. RO 4. NB |
| Jin Y 2012 | Unclear^1^ | Unclear^2^ | Unclear^2^ | Unclear^2^ | Unclear^2^ | Low^3^ | Unclear^4^ | 1.NR 2.NM 3. RO 4. NB |
| Jing GJ 2009 | Unclear^1^ | Unclear^2^ | Unclear^2^ | Unclear^2^ | Unclear^2^ | Low^3^ | Unclear^4^ | 1.NR 2.NM 3. RO 4. NB |
| Kuang JB 2008 | Low^1^ | Unclear^2^ | Unclear^2^ | Unclear^2^ | High^3^ | High^4^ | Unclear^5^ | 1.Using random number table generate the random sequence 2.NM 3. Reported 13 participants drop-out, but failed to describe the reasons. 4. Not reported all outcomes listed in the methods (6MWT) 5. NB |
| Li DW 2013 | Unclear^1^ | Unclear^2^ | Unclear^2^ | Unclear^2^ | Unclear^2^ | Low^3^ | Unclear^4^ | 1.NR 2.NM 3. RO 4. NB |
| Li GM 2011 | Unclear^1^ | Unclear^2^ | Unclear^2^ | Unclear^2^ | Unclear^2^ | Low^3^ | Unclear^4^ | 1.NR 2.NM 3. RO 4. NB |
| Li LC 2013 | Unclear^1^ | Unclear^2^ | Unclear^2^ | Unclear^2^ | Unclear^2^ | High^3^ | Unclear^4^ | 1.NR 2.NM 3. Not reported all outcomes listed in the methods (EF) 4. NB |
| Li P 2011 | Low^1^ | Unclear^2^ | Unclear^2^ | Unclear^2^ | Unclear^2^ | Low^3^ | Unclear^4^ | 1.Using random number table generate the random sequence 2.NM 3. RO 4. NB |
| Li Q 2014 | Unclear^1^ | Unclear^2^ | Low^3^ | Unclear^2^ | Unclear^2^ | Low^4^ | Unclear^5^ | 1.NR 2.NM 3. Reported the blinding methods 4. RO 5. NB |
| Li RY 2010a | Low^1^ | Unclear^2^ | Unclear^2^ | Unclear^2^ | Unclear^2^ | Low^3^ | Unclear^4^ | 1.Using random number table generate the random sequence 2.NM 3. RO 4. NB |
| Li SQ 2014 | Unclear^1^ | Unclear^2^ | Unclear^2^ | Unclear^2^ | Unclear^2^ | High^3^ | High^4^ | 1.NR 2.NM 3. Not reported all outcomes listed in the methods (LVEDV, ESV) 4.Not reported comparability of baseline data and sample size calculation. |
| Li SZ 2009 | Unclear^1^ | Unclear^2^ | Unclear^2^ | Unclear^2^ | Unclear^2^ | High^3^ | Unclear^4^ | 1.NR 2.NM 3. Not reported all outcomes listed in the methods (echocardiography) 4. NB |
| Li T 2010 | Unclear^1^ | Unclear^2^ | Unclear^2^ | Unclear^2^ | Unclear^2^ | Low^3^ | Unclear^4^ | 1.NR 2.NM 3. RO 4. NB |
| Li WY 2013 | Low^1^ | Unclear^2^ | Unclear^2^ | Unclear^2^ | Unclear^2^ | High^3^ | Unclear^4^ | 1.Using random number table generate the random sequence 2.NM 3. Not reported all outcomes listed in the methods (Electrocardiograph) 4. NB |
| Li XL 2013 | Low | Low | Low | Low | Low | Low | Low | This trial were registered at Chinese Clinical Trial Registry, and it reported random methods and allocation concealment, and they used blind methods for participants and personnels. This trial reported comparability of baseline data and sample size calculation. |
| Li YH 2013 | Unclear^1^ | Unclear^2^ | Unclear^2^ | Unclear^2^ | Unclear^2^ | High^3^ | Unclear^4^ | 1.NR 2.NM 3. Not reported all outcomes listed in the methods (LVEDV, LVESV) 4. NB |
| Li YX 2012 | Unclear^1^ | Unclear^2^ | Unclear^2^ | Unclear^2^ | Unclear^3^ | Low^4^ | Unclear^5^ | 1.NR 2.NM 3.The author did not reported if there was attrition in trial as well as PP or ITT analysis. Insufficient information to judge. 4. RO 5. NB |
| Li YX 2013 | Unclear^1^ | Unclear^2^ | Unclear^2^ | Unclear^2^ | Unclear^2^ | Low^3^ | Unclear^4^ | 1.NR 2.NM 3. RO 4. NB |
| Lin JH 2008 | Unclear^1^ | Unclear^2^ | Unclear^2^ | Unclear^2^ | Unclear^2^ | Unclear^2^ | Unclear^3^ | 1.NR 2.NM 3. NB |
| Lin ZJ 2010 | Unclear^1^ | Unclear^2^ | Unclear^2^ | Unclear^2^ | Unclear^2^ | High^3^ | Unclear^4^ | 1.NR 2.NM 3. Not reported all outcomes listed in the methods (electrocardiogram, electrolyte) 4. NB |
| Liu HL 2008 | Unclear^1^ | Unclear^2^ | Unclear^2^ | Unclear^2^ | Unclear^2^ | Low^3^ | Unclear^4^ | 1.NR 2.NM 3. RO 4. NB |
| Liu J 2008 | Unclear^1^ | Unclear^2^ | Unclear^2^ | Unclear^2^ | Unclear^2^ | Low^3^ | Unclear^4^ | 1.NR 2.NM 3. RO 4. NB |
| Liu LX 2014 | Unclear^1^ | Unclear^2^ | Unclear^2^ | Unclear^2^ | Unclear^2^ | Low^3^ | Unclear^4^ | 1.NR 2.NM 3. RO 4. NB |
| Liu SJ 2009 | Unclear^1^ | Unclear^2^ | Unclear^2^ | Unclear^2^ | Unclear^2^ | High^3^ | Unclear^4^ | 1.NR 2.NM 3. Not reported all outcomes listed in the methods (LVEDV) 4. NB |
| Liu T 2013 | Low^1^ | Unclear^2^ | Unclear^2^ | Unclear^2^ | Unclear^2^ | Low^3^ | Unclear^4^ | 1.Using random number table generate the random sequence 2.NM 3. RO 4. NB |
| Liu TR 2010 | Unclear^1^ | Unclear^2^ | Unclear^2^ | Unclear^2^ | Unclear^2^ | Low^3^ | Unclear^4^ | 1.NR 2.NM 3. RO 4. NB |
| Liu WJ 2007 | Unclear^1^ | Unclear^2^ | Unclear^2^ | Unclear^2^ | Unclear^2^ | Low^3^ | High^4^ | 1.NR 2.NM 3. RO 4.Not reported comparability of baseline data and sample size calculation. |
| Liu XC 2008 | Unclear^1^ | Unclear^2^ | Unclear^2^ | Unclear^2^ | Unclear^2^ | Low^3^ | Unclear^4^ | 1.NR 2.NM 3. RO 4. NB |
| Liu XC 2011 | Unclear^1^ | Unclear^2^ | Unclear^2^ | Unclear^2^ | Unclear^2^ | Low^3^ | Unclear^4^ | 1.NR 2.NM 3. RO 4. NB |
| Liu XG 2013 | Low^1^ | Unclear^2^ | Unclear^2^ | Unclear^2^ | Unclear^2^ | Low^3^ | Unclear^4^ | 1.Using random number table generate the random sequence 2.NM 3. RO 4. NB |
| Liu XM 2010 | Unclear^1^ | Unclear^2^ | Unclear^2^ | Unclear^2^ | Unclear^2^ | Low^3^ | Unclear^4^ | 1.NR 2.NM 3. RO 4. NB |
| Liu XM 2013 | Unclear^1^ | Unclear^2^ | Unclear^2^ | Unclear^2^ | Unclear^2^ | Low^3^ | Unclear^4^ | 1.NR 2.NM 3. RO 4. NB |
| Liu YJ 2012 | Unclear^1^ | Unclear^2^ | Unclear^2^ | Unclear^2^ | Unclear^2^ | High^3^ | Unclear^4^ | 1.NR 2.NM 3. Not reported all outcomes listed in the methods (HR, ADR, urine volume) 4. NB |
| Long F 2009 | Unclear^1^ | Unclear^2^ | Unclear^2^ | Unclear^2^ | Unclear^2^ | Low^3^ | Unclear^4^ | 1.NR 2.NM 3. RO 4. NB |
| Lu JP 2012 | Unclear^1^ | Unclear^2^ | Unclear^2^ | Unclear^2^ | Unclear^2^ | High^3^ | Unclear^4^ | 1.NR 2.NM 3. Not reported all outcomes listed in the methods (BP, HR) 4. NB |
| Luo Q 2013 | Unclear^1^ | Unclear^2^ | Unclear^2^ | Unclear^2^ | Unclear^2^ | Low^3^ | Unclear^4^ | 1.NR 2.NM 3. RO 4. NB |
| Ma AP 2013 | Unclear^1^ | Unclear^2^ | Unclear^2^ | Unclear^2^ | Unclear^2^ | Unclear^2^ | Unclear^3^ | 1.NR 2.NM 3. NB |
| Ma FF 2008a1 & Ma FF 2008a2 | Low^1^ | Unclear^2^ | Unclear^2^ | Unclear^2^ | Unclear^2^ | Low^3^ | Unclear^4^ | 1.Using random number table generate the random sequence 2.NM 3. RO 4. NB |
| Ma FF 2008b | Low^1^ | Unclear^2^ | Unclear^2^ | Unclear^2^ | Unclear^2^ | Low^3^ | Unclear^4^ | 1.Using random number table generate the random sequence 2.NM 3. RO 4. NB |
| Ma L 2010 | Unclear^1^ | Unclear^2^ | Unclear^2^ | Unclear^2^ | Unclear^2^ | Low^3^ | Unclear^4^ | 1.NR 2.NM 3. RO 4. NB |
| Ma RX 2014 | Unclear^1^ | Unclear^2^ | Unclear^2^ | Unclear^2^ | Unclear^2^ | Low^3^ | Unclear^4^ | 1.NR 2.NM 3. RO 4. NB |
| Miao S 2013 | Unclear^1^ | Unclear^2^ | Unclear^2^ | Unclear^2^ | Unclear^2^ | Low^3^ | Unclear^4^ | 1.NR 2.NM 3. RO 4. NB |
| Niu LY 2012 | Unclear^1^ | Unclear^2^ | Unclear^2^ | Unclear^2^ | Unclear^2^ | Low^3^ | Unclear^4^ | 1.NR 2.NM 3. RO 4. NB |
| Pang XM 2008 | Unclear^1^ | Unclear^2^ | Unclear^2^ | Unclear^2^ | Unclear^2^ | High^3^ | High^4^ | 1.NR 2.NM 3. Not reported all outcomes listed in the methods (HR, BP, CO, CI, EF) 4.Not reported comparability of baseline data and sample size calculation. |
| Qiu X 2013 | Unclear^1^ | Unclear^2^ | Unclear^2^ | Unclear^2^ | Unclear^2^ | Low^3^ | Unclear^4^ | 1.NR 2.NM 3. RO 4. NB |
| Rao LZ 2012 | Unclear^1^ | Unclear^2^ | Unclear^2^ | Unclear^2^ | Unclear^2^ | Low^3^ | Unclear^4^ | 1.NR 2.NM 3. RO 4. NB |
| Shen R 2010 | Unclear^1^ | Unclear^2^ | Unclear^2^ | Unclear^2^ | Unclear^2^ | Low^3^ | Unclear^4^ | 1.NR 2.NM 3. RO 4. NB |
| Shen XR 2014 | Unclear^1^ | Unclear^2^ | Unclear^2^ | Unclear^2^ | Unclear^2^ | Low^3^ | Unclear^4^ | 1.NR 2.NM 3. RO 4. NB |
| Shi CP 2013 | Unclear^1^ | Unclear^2^ | Unclear^2^ | Unclear^2^ | Unclear^2^ | Low^3^ | Unclear^4^ | 1.NR 2.NM 3. RO 4. NB |
| Su HM 2007 | Low^1^ | Unclear^2^ | Unclear^2^ | Unclear^2^ | Unclear^2^ | Low^3^ | Unclear^4^ | 1.Using random number table generate the random sequence 2.NM 3. RO 4. NB |
| Su LJ 2012 | Unclear^1^ | Unclear^2^ | Unclear^2^ | Unclear^2^ | Unclear^2^ | Low^3^ | Unclear^4^ | 1.NR 2.NM 3. RO 4. NB |
| Su RY 2013 | Unclear^1^ | Unclear^2^ | Unclear^2^ | Unclear^2^ | Unclear^2^ | Low^3^ | Unclear^4^ | 1.NR 2.NM 3. RO 4. NB |
| Sun LP 2007 | Unclear^1^ | Unclear^2^ | Unclear^2^ | Unclear^2^ | Low^3^ | Low^4^ | Unclear^5^ | 1.NR 2.NM 3. The author committed in the article that no person drop out, fall off or loss of follow up in text 4. Reported all outcomes listed in the methods 5. NB |
| Tang SY 2013 | Unclear^1^ | Unclear^2^ | Unclear^2^ | Unclear^2^ | Unclear^2^ | Low^3^ | Unclear^4^ | 1.NR 2.NM 3. RO 4. NB |
| Tao X 2011 | Unclear^1^ | Unclear^2^ | Unclear^2^ | Unclear^2^ | Unclear^2^ | Low^3^ | Unclear^4^ | 1.NR 2.NM 3. RO 4. NB |
| Tian Y 2011 | Unclear^1^ | Unclear^2^ | Unclear^2^ | Unclear^2^ | Unclear^2^ | Low^3^ | Unclear^4^ | 1.NR 2.NM 3. RO 4. NB |
| Wang N 2014 | Unclear^1^ | Unclear^2^ | Unclear^2^ | Unclear^2^ | Unclear^2^ | Unclear^2^ | Unclear^3^ | 1.NR 2.NM 3. NB |
| Wang Q 2012 | Unclear^1^ | Unclear^2^ | Unclear^2^ | Unclear^2^ | Unclear^2^ | Low^3^ | Unclear^4^ | 1.NR 2.NM 3. RO 4. NB |
| Wang SZ 2012 | Unclear^1^ | Unclear^2^ | Unclear^2^ | Unclear^2^ | Unclear^2^ | Low^3^ | Unclear^4^ | 1.NR 2.NM 3. RO 4. NB |
| Wang YY 2013 | Unclear^1^ | Unclear^2^ | Unclear^2^ | Unclear^2^ | Unclear^2^ | Low^3^ | Unclear^4^ | 1.NR 2.NM 3. RO 4. NB |
| Wei XB 2013 | Unclear^1^ | Unclear^2^ | Unclear^3^ | Unclear^2^ | Unclear^2^ | Low^3^ | Unclear^4^ | 1.NR 2.NM 3. RO 4. NB |
| Wen Y 2012 | Unclear^1^ | Unclear^2^ | Unclear^2^ | Unclear^2^ | Unclear^2^ | Low^3^ | Unclear^4^ | 1.NR 2.NM 3. RO 4. NB |
| Wu GL 2015 | Unclear^1^ | Unclear^2^ | Unclear^2^ | Unclear^2^ | Unclear^2^ | Low^3^ | Unclear^4^ | 1.NR 2.NM 3. RO 4. NB |
| Wu X 2014 | Low^1^ | Unclear^2^ | Unclear^2^ | Unclear^2^ | Unclear^2^ | Low^3^ | Unclear^4^ | 1.Using random number table generate the random sequence 2.NM 3. RO 4. NB |
| Wu SP 2014 | Unclear^1^ | Unclear^2^ | Unclear^2^ | Unclear^2^ | Unclear^2^ | Low^3^ | Unclear^4^ | 1.NR 2.NM 3. RO 4. NB |
| Xiong SQ 2014 | Low^1^ | Unclear^2^ | Unclear^2^ | Unclear^2^ | Unclear^2^ | Low^3^ | Unclear^4^ | 1.Using random number table generate the random sequence 2.NM 3. RO 4. NB |
| Xu GS 2014 | Unclear^1^ | Unclear^2^ | Unclear^2^ | Unclear^2^ | Unclear^2^ | Low^3^ | Unclear^4^ | 1.NR 2.NM 3. RO 4. NB |
| Xue L 2014 | Unclear^1^ | Unclear^2^ | Unclear^2^ | Unclear^2^ | Unclear^2^ | Low^3^ | Unclear^4^ | 1.NR 2.NM 3. RO 4. NB |
| Xue LX 2008 | Unclear^1^ | Unclear^2^ | Unclear^2^ | Unclear^2^ | Unclear^2^ | Low^3^ | Unclear^4^ | 1.NR 2.NM 3. RO 4. NB |
| Yan KL 2012 | Unclear^1^ | Unclear^2^ | Unclear^2^ | Unclear^2^ | Unclear^2^ | Low^3^ | Unclear^4^ | 1.NR 2.NM 3. RO 4. NB |
| Yang DK 2014 | Unclear^1^ | Unclear^2^ | Unclear^2^ | Unclear^2^ | Unclear^2^ | Low^3^ | Unclear^4^ | 1.NR 2.NM 3. RO 4. NB |
| Yang F 2007 | Unclear^1^ | Unclear^2^ | Unclear^3^ | Unclear^3^ | Unclear^3^ | Low^4^ | Unclear^5^ | 1.NR 2.Using an envelope but failed to provide further details 3.NM 4. RO 5. NB |
| Yang HT 2012 | Unclear^1^ | Unclear^2^ | Unclear^2^ | Unclear^2^ | Unclear^2^ | Low^3^ | Unclear^4^ | 1.NR 2.NM 3. RO 4. NB |
| Yang HT 2013 | Unclear^1^ | Unclear^2^ | Unclear^2^ | Unclear^2^ | Unclear^2^ | High^3^ | Unclear^4^ | 1.NR 2.NM 3. Not reported all outcomes listed in the methods (HR, BP) 4. NB |
| Yang J 2013 | Low^1^ | Unclear^2^ | Unclear^2^ | Unclear^2^ | Unclear^2^ | High^3^ | Unclear^4^ | 1.Using random number table generate the random sequence 2.NM 3. Not reported all outcomes listed in the methods (rehospitalization rate, case fatality rate) 4. NB |
| Yang W 2012 | Unclear^1^ | Unclear^2^ | Unclear^2^ | Unclear^2^ | Unclear^2^ | Low^3^ | Unclear^4^ | 1.NR 2.NM 3. RO 4. NB |
| Yao L 2011 | Unclear^1^ | Unclear^2^ | Unclear^2^ | Unclear^2^ | Unclear^2^ | Low^3^ | Unclear^4^ | 1.NR 2.NM 3. RO 4. NB |
| Ye RS 2013 | Unclear^1^ | Unclear^2^ | Unclear^2^ | Unclear^2^ | Unclear^2^ | Low^3^ | Unclear^4^ | 1.NR 2.NM 3. RO 4. NB |
| Ye S 2012 | Unclear^1^ | Unclear^2^ | Unclear^2^ | Unclear^2^ | Unclear^2^ | Low^3^ | Unclear^4^ | 1.NR 2.NM 3. RO 4. NB |
| Yin ZL 2009 | Unclear^1^ | Unclear^2^ | Unclear^2^ | Unclear^2^ | Unclear^2^ | Low^3^ | High^4^ | 1.NR 2.NM 3. RO 4.Not reported comparability of baseline data and sample size calculation. |
| Ying M 2013 | Unclear^1^ | Unclear^2^ | Unclear^2^ | Unclear^2^ | Unclear^2^ | Low^3^ | Unclear^4^ | 1.NR 2.NM 3. RO 4. NB |
| Yu JH 2008 | Unclear^1^ | Unclear^2^ | Low^3^ | Unclear^2^ | Unclear^2^ | Low^4^ | Unclear^5^ | 1.NR 2.NM 3. Using double-blinded, placebo-controlled design 4. RO 5. NB |
| Yuan JK 2012 | Unclear^1^ | Unclear^2^ | Unclear^2^ | Unclear^2^ | Unclear^2^ | Low^3^ | Unclear^4^ | 1.NR 2.NM 3. RO 4. NB |
| Zhai N 2015 | Unclear^1^ | Unclear^2^ | Unclear^2^ | Unclear^2^ | Unclear^2^ | Low^3^ | Unclear^4^ | 1.NR 2.NM 3. RO 4. NB |
| Zhang CA 2013 | Unclear^1^ | Unclear^2^ | Unclear^2^ | Unclear^2^ | Unclear^2^ | Low^3^ | Unclear^4^ | 1.NR 2.NM 3. RO 4. NB |
| Zhang H 2011 | Unclear^1^ | Unclear^2^ | Unclear^2^ | Unclear^2^ | Unclear^2^ | High^3^ | High^4^ | 1.NR 2.NM 3. Not reported all outcomes listed in the methods (blood routine, urine routine, cardiac ultrasound) 4.Not reported comparability of baseline data and sample size calculation. |
| Zhang J 2015 | Unclear^1^ | Unclear^2^ | Unclear^2^ | Unclear^2^ | Unclear^2^ | Low^3^ | Unclear^4^ | 1.NR 2.NM 3. RO 4. NB |
| Zhang R 2014 | Unclear^1^ | Unclear^2^ | Unclear^2^ | Unclear^2^ | Unclear^2^ | Low^3^ | Unclear^4^ | 1.NR 2.NM 3. RO 4. NB |
| Zhang WL 2013 | Unclear^1^ | Unclear^2^ | Unclear^2^ | Unclear^2^ | Unclear^2^ | Low^3^ | Unclear^4^ | 1.NR 2.NM 3. RO 4. NB |
| Zhang XX 2010 | Unclear^1^ | Unclear^2^ | Unclear^2^ | Unclear^2^ | Unclear^2^ | High^3^ | Unclear^4^ | 1.NR 2.NM 3. Not reported all outcomes listed in the methods (LVEDd, 6MWT) 4. NB |
| Zhao JS 2014 | Low^1^ | Unclear^2^ | Unclear^2^ | Unclear^2^ | Unclear^2^ | High^3^ | Unclear^4^ | 1.Using random number table generate the random sequence 2.NM 3. Not reported all outcomes listed in the methods (rehospitalization rate, case fatality rate, cardiovascular events) 4. NB |
| Zhao MJ 2009 & Zhao MJ 2012 | Unclear^1^ | Unclear^2^ | Unclear^2^ | Unclear^2^ | Unclear^2^ | Low^3^ | Unclear^4^ | 1.NR 2.NM 3. RO 4. NB |
| Zheng JJ 2012 | Low^1^ | Unclear^2^ | Unclear^2^ | Unclear^2^ | Unclear^2^ | Low^3^ | Unclear^4^ | 1.Using random number table generate the random sequence 2.NM 3. RO 4. NB |
| Zheng LW 2013 | Unclear^1^ | Unclear^2^ | Unclear^2^ | Unclear^2^ | Unclear^2^ | High^3^ | Unclear^4^ | 1.NR 2.NM 3. Not reported all outcomes listed in the methods (LVESD) 4. NB |
| Zheng WH 2014 | Unclear^1^ | Unclear^2^ | Unclear^2^ | Unclear^2^ | Unclear^2^ | Low^3^ | Unclear^4^ | 1.NR 2.NM 3. RO 4. NB |
| Zhou FZ 2011a | Unclear^1^ | Unclear^2^ | Unclear^2^ | Unclear^2^ | Unclear^2^ | Low^3^ | Unclear^4^ | 1.NR 2.NM 3. RO 4. NB |
| Zhou Y 2013 | Unclear^1^ | Unclear^2^ | Unclear^2^ | Unclear^2^ | Unclear^2^ | Low^3^ | Unclear^4^ | 1.NR 2.NM 3. RO 4. NB |
| Zhu HG 2012 | Unclear^1^ | Unclear^2^ | Unclear^2^ | Unclear^2^ | Unclear^2^ | Low^3^ | Unclear^4^ | 1.NR 2.NM 3. RO 4. NB |
| Zhuo JY 2013 | Unclear^1^ | Unclear^2^ | Unclear^2^ | Unclear^2^ | Unclear^2^ | Low^3^ | Unclear^4^ | 1.NR 2.NM 3. RO 4. NB |

NR: No specific randomisation method reported in text

NM: Not mention this item in text

RO: Authors reported all outcomes listed in the methods

NB: Authors reported comparability of baseline data, however failed to report sample size calculation.

LVEDd: Left ventricular end-diastolic dimension

LVESD: Left ventricular end-systolic diameter

LVEDV:Left ventricular end-diastolic volume

LVESV: Left ventricular end systolic volume

LVDs: Low Voltage Differential Signaling

ESV: end-systolic volume
