# Supplementary material for: Clinical effects of a standardized Chinese herbal remedy, Qili Qiangxin, as an adjuvant treatment in heart failure: systematic review and meta-analysis
Source: BMC Complement Altern Med. 2016 Jul 11;16:201. doi: 10.1186/s12906-016-1174-1 (PMC4940829; doi:10.1186/s12906-016-1174-1)
Supplement: Additional file 4: — Forest plots for all meta-analyses. (DOC 660 kb) [file 12906_2016_1174_MOESM4_ESM.doc]

**Additional file 4. Forest plots for all meta-analyses**

1. **Qili Qiangxin capsule plus conventional treatment versus conventional treatment**
   1. **Outcome: All-cause mortality or cardiovascular mortality**


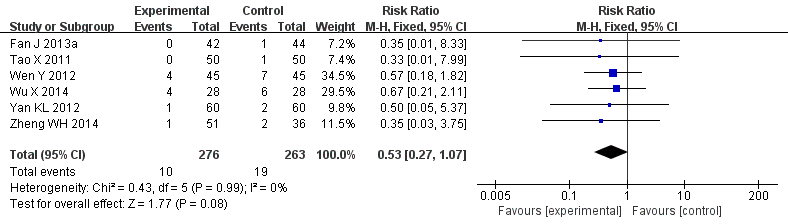


Figure 1.

- 1. **Outcome: Major cardiovascular events**


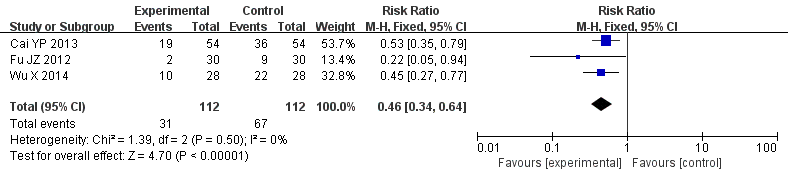


Figure 2.

- 1. **Outcome: Hospitalizations due to heart failure**


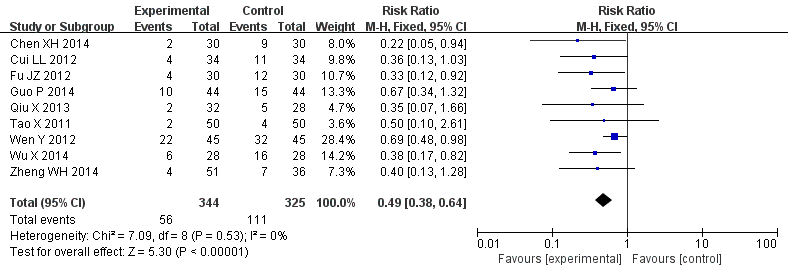


Figure 3.

- 1. **Outcome: cardiac function (defined as an increase of two or more functional classes using NYHA)**


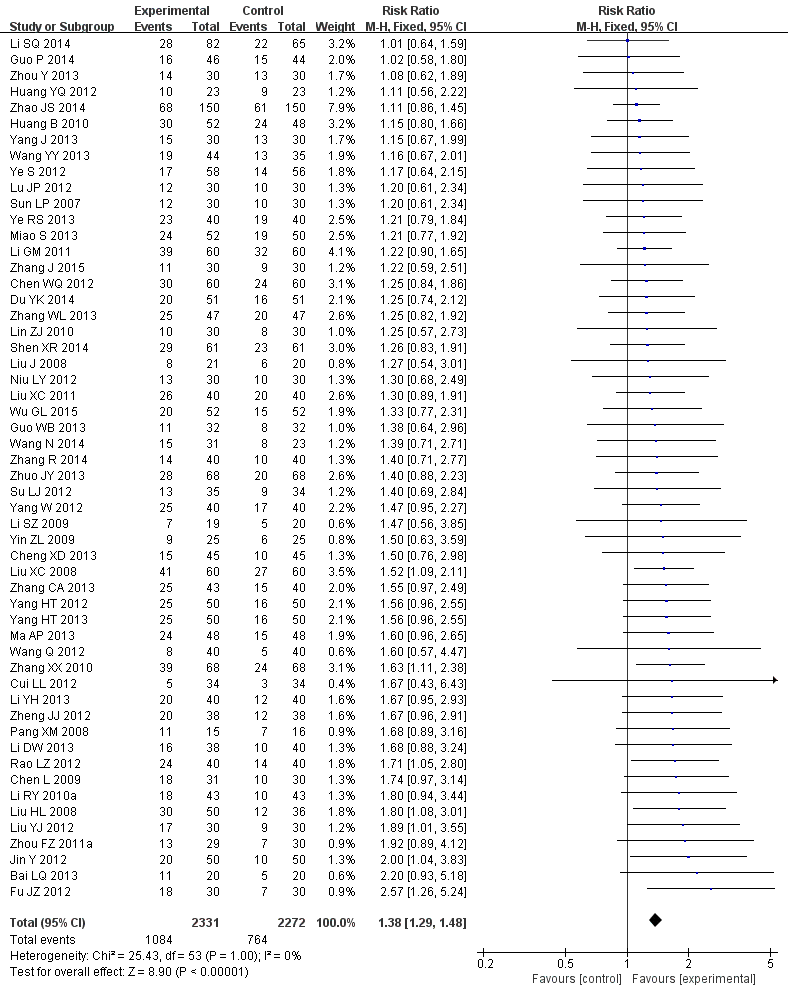


Figure 4.

- 1. **Outcome: Quality of life (QOL)**


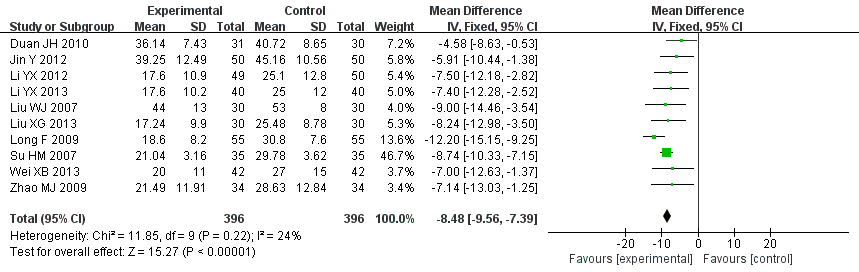


Figure 5.

- 1. **Outcome: Adverse drug reactions (ADR)**


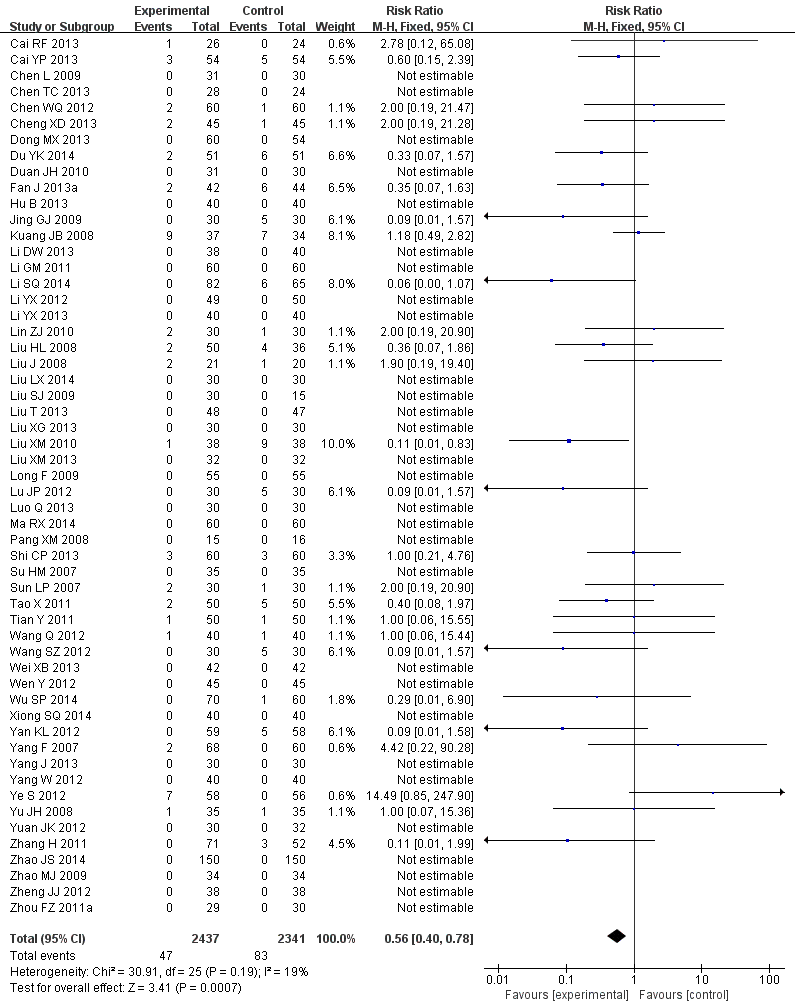


Figure 6.

- 1. **Outcome: Left ventricular ejection fraction (LVEF)**


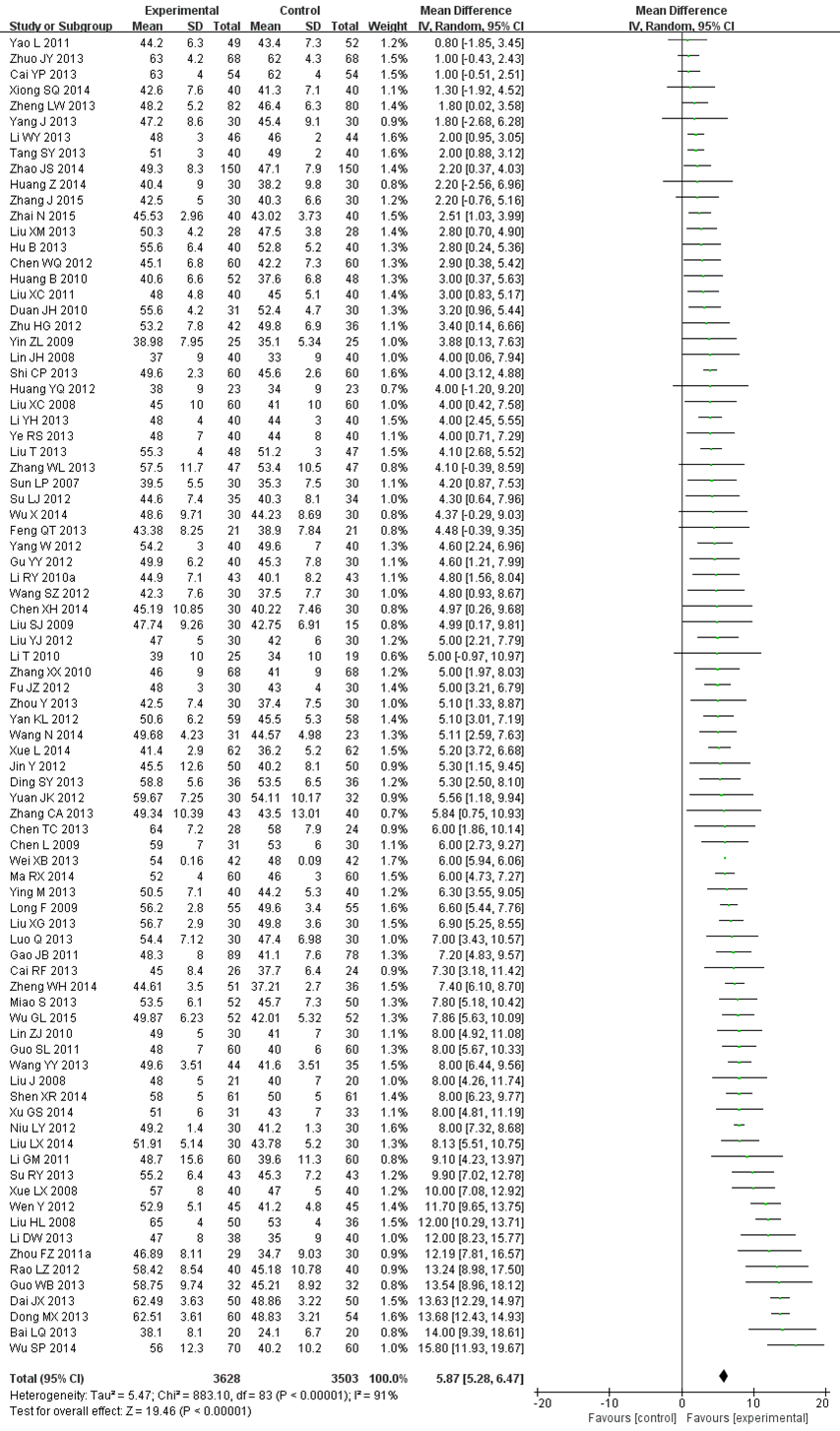


Figure 7.

**Subgroup analyses on treatment duration:**


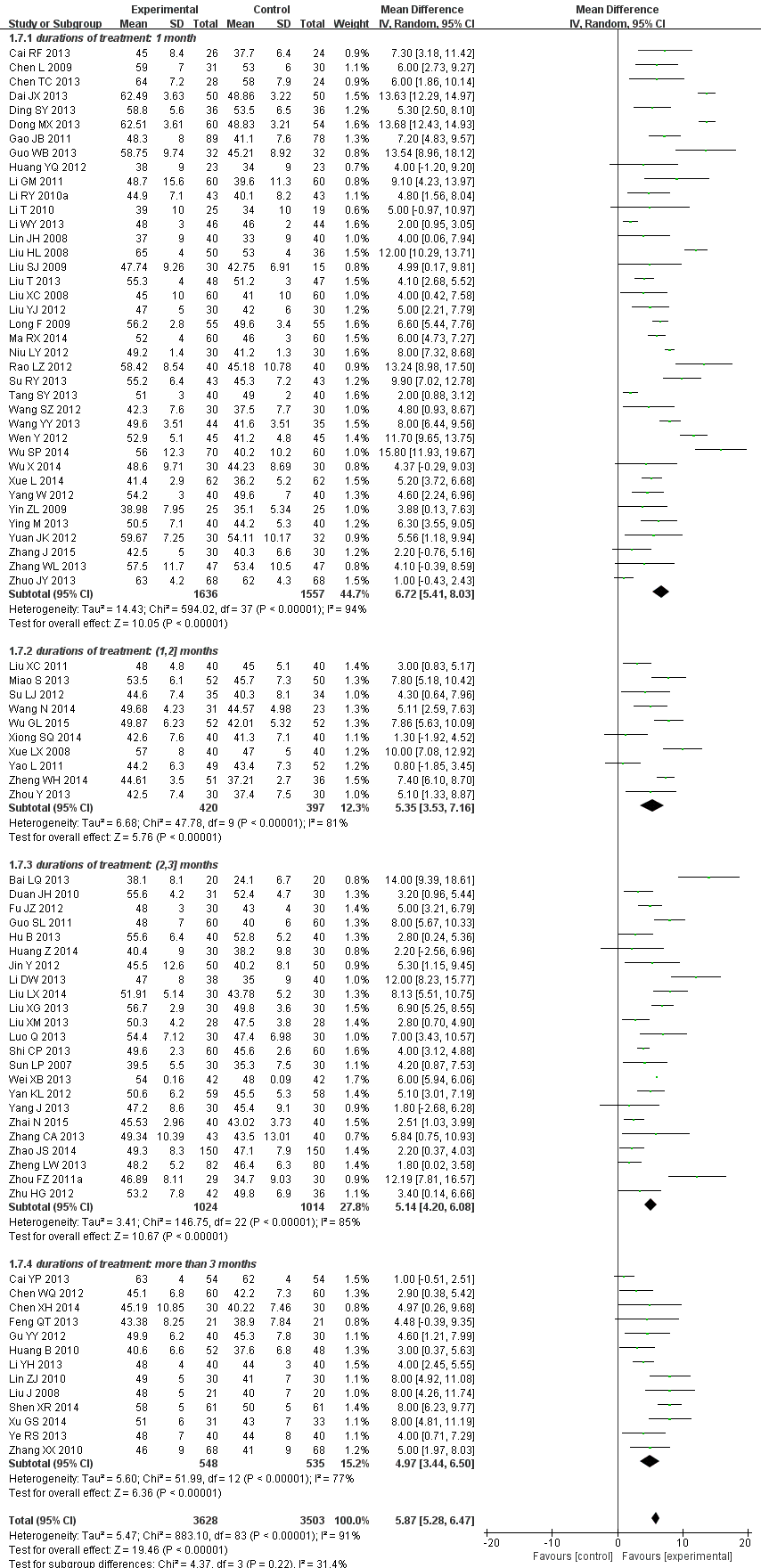


Figure 8.

- 1. **Outcome: Plasma amino-terminal pro-brain natriuretic peptide (NT-proBNP)**


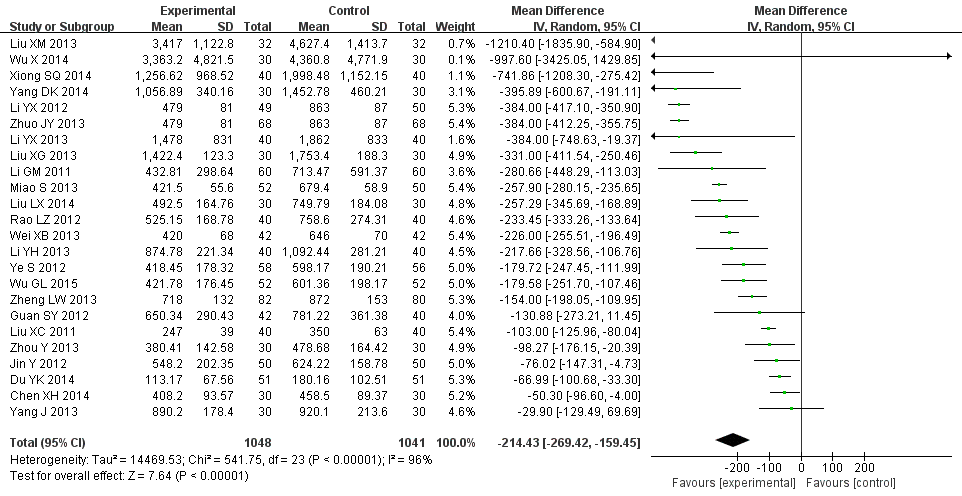


Figure 9.

- 1. **Outcome: Six-minutes walking distance (6MWD)**


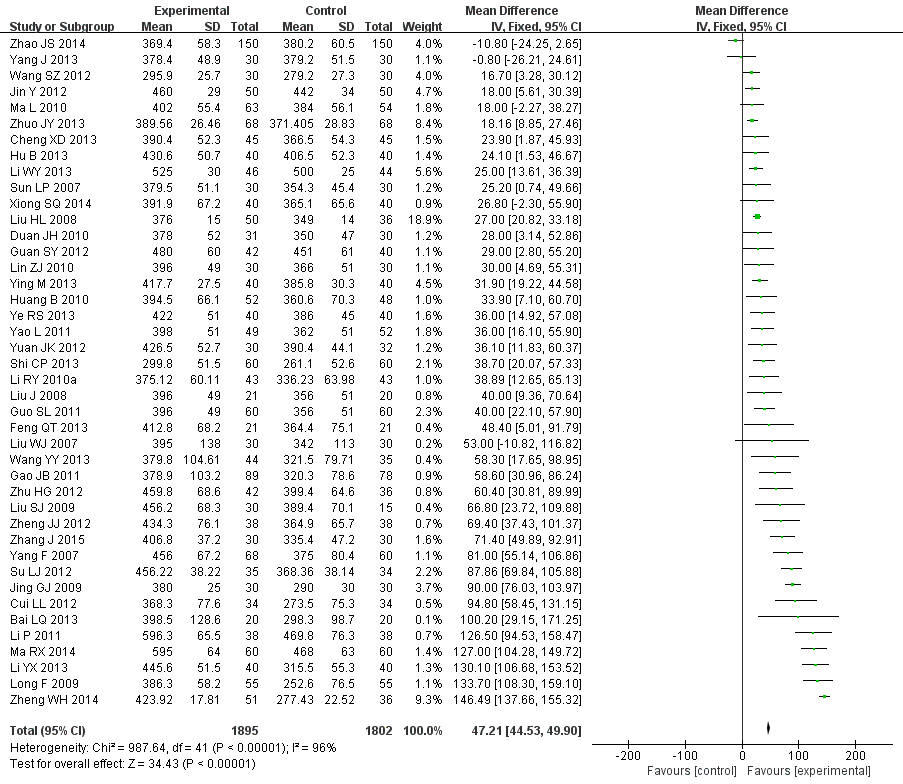


Figure 10.

1. **Qili Qiangxin capsule plus conventional treatment versus placebo plus conventional treatment**
   1. **Outcome: Six-minutes walking distance (6MWD)**


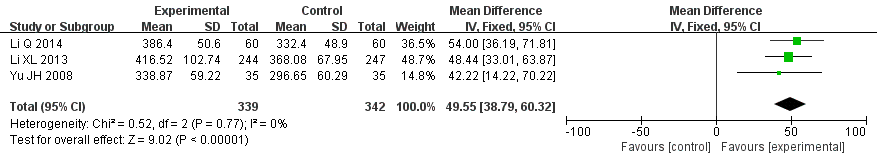


Figure 11.

1. **Qili Qiangxin capsule plus** **conventional treatment versus medications recommended in guidelines plus conventional treatment**
   1. **Outcome: cardiac function**


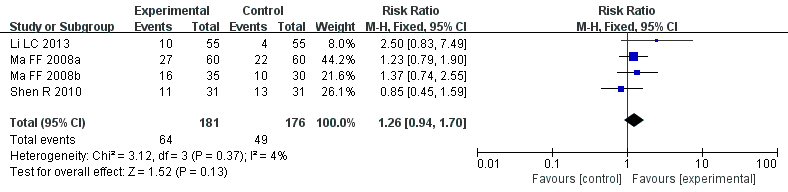


Figure 12.

- 1. **Outcome: cardiac function ---subgroup analyses**


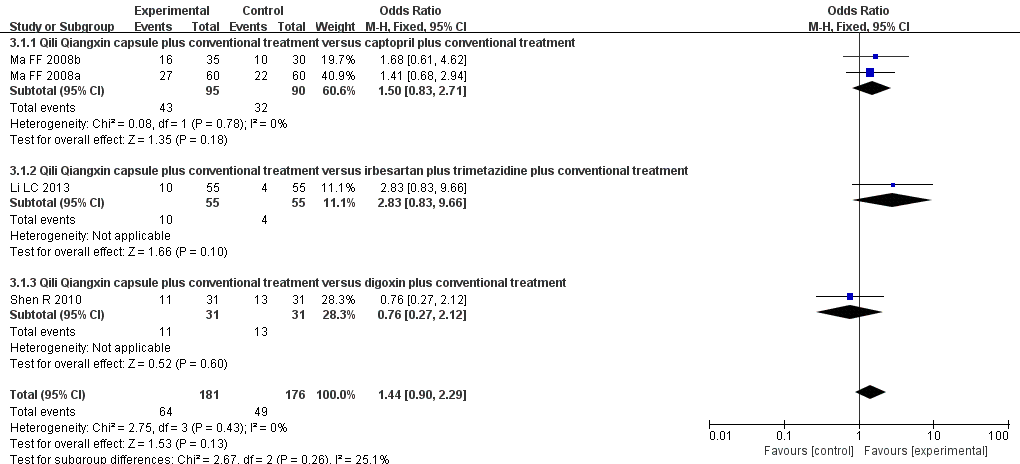


Figure 13

- 1. **Outcome: Adverse drug reactions (ADR)**


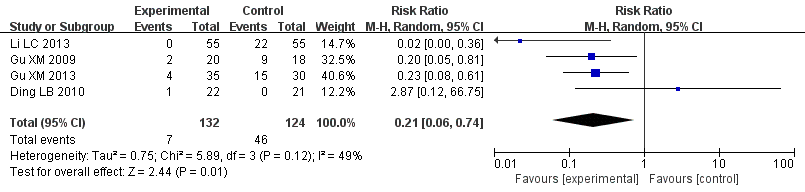


Figure 14

- 1. **Outcome: Six-minutes walking distance (6MWD)**


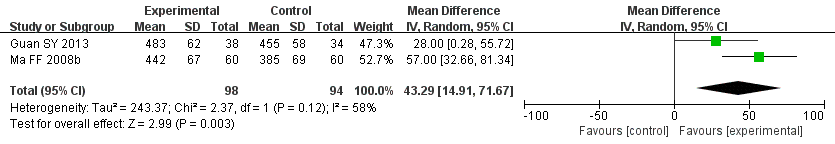


Figure 15
